# Nrf2 overexpression rescues the RPE in mouse models of retinitis pigmentosa

**DOI:** 10.1172/jci.insight.145029

**Published:** 2021-01-25

**Authors:** David M. Wu, Xuke Ji, Maryna V. Ivanchenko, Michelle Chung, Mary Piper, Parimal Rana, Sean K. Wang, Yunlu Xue, Emma West, Sophia R. Zhao, Hongbin Xu, Marcelo Cicconet, Wenjun Xiong, Constance L. Cepko

**Affiliations:** 1 Massachusetts Eye and Ear Infirmary Retina Service, Department of Ophthalmology,; 2 Departments of Genetics and Ophthalmology, Blavatnik Institute, and; 3 Department of Neurobiology, Harvard Medical School, Boston, Massachusetts, USA.; 4 Howard Hughes Medical Institute, Chevy Chase, Maryland, USA.; 5 Department of Bioinformatics, T.H. Chan Harvard School of Public Health, Boston, Massachusetts, USA.; 6 Image and Data Analysis Core, Harvard Medical School, Boston, Massachusetts, USA.; 7 Department of Biomedical Sciences, City University of Hong Kong, Hong Kong, China.

**Keywords:** Ophthalmology, Gene therapy

## Abstract

Nrf2, a transcription factor that regulates the response to oxidative stress, has been shown to rescue cone photoreceptors and slow vision loss in mouse models of retinal degeneration (rd). The retinal pigment epithelium (RPE) is damaged in these models, but whether it also could be rescued by Nrf2 has not been previously examined. We used an adeno-associated virus (AAV) with an RPE-specific (Best1) promoter to overexpress Nrf2 in the RPE of rd mice. Control rd mice showed disruption of the regular array of the RPE, as well as loss of RPE cells. Cones were lost in circumscribed regions within the cone photoreceptor layer. Overexpression of Nrf2 specifically in the RPE was sufficient to rescue the RPE, as well as the disruptions in the cone photoreceptor layer. Electron microscopy showed compromised apical microvilli in control rd mice but showed preserved microvilli in Best1-Nrf2–treated mice. The rd mice treated with Best1-Nrf2 had slightly better visual acuity. Transcriptome profiling showed that Nrf2 upregulates multiple oxidative defense pathways, reversing declines seen in the glutathione pathway in control rd mice. In summary, Nrf2 overexpression in the RPE preserves RPE morphology and survival in rd mice, and it is a potential therapeutic for diseases involving RPE degeneration, including age-related macular degeneration (AMD).

## Introduction

One of the challenges in understanding retinitis pigmentosa is how mutations leading to the death of a particular cell type then lead to a broader degeneration. Mutations in a rod-specific phosphodiesterase, *Pde6b*, lead to the death of rods (1, 2). Cones, which do not express this phosphodiesterase, die only after the death of the rods. Because this pattern is recapitulated across many other genetic etiologies of retinitis pigmentosa, which similarly harbor mutations in genes expressed only in rods, it seems that the absence of rods leads to cone death (3). A contributing factor to cone death has been proposed to be a hyperoxic environment (4, 5). The retinal pigment epithelium (RPE) and portions of the retina normally experience a high-oxygen environment, which, in RP, is exacerbated by the loss of rods, as rods consume a significant amount of oxygen (6). As rods are lost, oxygen continues to flow into the outer retina from the choriocapillaris, creating a hyperoxic environment that is presumably hostile for the remaining cells. One protective strategy is, thus, to augment the oxidative damage defense systems. Our group recently created an adeno-associated virus (AAV) vector that overexpresses Nrf2, a transcription factor that not only regulates antioxidant defense pathways, but also antiinflammatory and xenobiotic pathways. When tested in mouse models of RP, it led to greater survival of cones and retention of vision in all 3 mouse models of RP that were tested (7). Similarly, reduction in a negative regulatory pathway for Nrf2 led to greater visual system function in an acute oxidative damage model (8), and treatment with antioxidants in a retinal degeneration (rd) model was shown to preserve cones and cone-mediated vision (9). In our previous experiments, a CMV promoter was used to drive Nrf2. As the CMV promoter is strongly expressed in cones as well as the RPE, it was not clear if the benefits were due to expression in cones, in the RPE, or in both cell types. In addition, our previous study did not analyze the effects of this vector on the RPE.

Migration of the RPE into the retina of retinitis pigmentosa patients has long been observed to manifest as bone spicule pigmentation (10). Although less studied, the RPE in mice with *rd* mutations also has been observed to undergo significant morphological changes (11, 12). The significance of these changes to the survival of photoreceptors is unknown. It seems reasonable to assume, given that the RPE and retina have a symbiotic relationship, that RPE stress could contribute to the degeneration of cones (13). We wished to address these points by overexpressing Nrf2 specifically in the RPE and then assessing its ability to protect the RPE, as well as to see what effects this may have on the retina.

## Results

**Figure 1:**
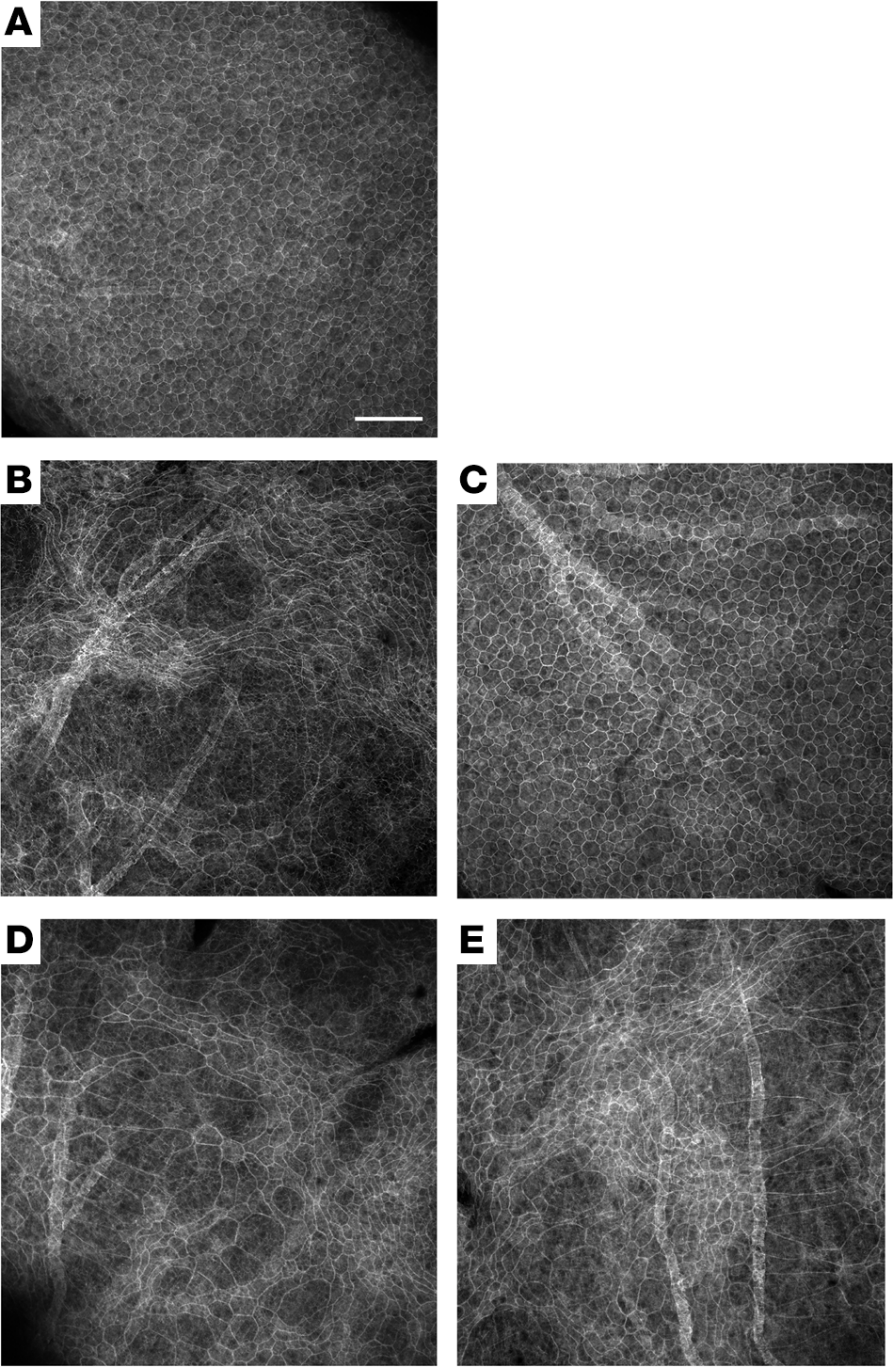
Morphological analyses of the RPE from WT mice and *rd1* mice with or without AAV-Best1-Nrf2. (**A**) An RPE flat mount from a P42 WT (CD1) mouse is shown. Phalloidin (gray) stains the F-actin belt around the circumference of individual RPE cells and shows the regular hexagonal array of this epithelial monolayer. (**B**) An RPE flat mount from a P42 *rd1* mouse that received an injection only of control virus (AAV-hRedO-H2b-GFP) at P0. (**C**) The RPE flat mount from the other eye of the same P42 *rd1* mouse as shown in **B**, following injection with AAV-Best1-Nrf2 plus AAV-hRedO-H2b-GFP. (**D**) An RPE flat mount of a P42 *rd1* mouse that received an injection of control virus (AAV-hRedO-H2b-GFP). (**E**) The RPE flat mount from the other eye of the same mouse from **D**, which received an injection of AAV-hRedO-Nrf2 plus AAV-hRedO-H2b-GFP at P0. Panels are representative of findings from *n* > 20 mice per group. Scale bar: 100 μm.

*Nrf2overexpressionintheRPEisnecessaryandsufficienttopreventdegenerationoftheRPE*. The RPE is typically a hexagonal monolayer of epithelial cells located between the retina and the choroid (Figure 1A). The loss of rods in retinitis pigmentosa leads to collateral stress and death in the RPE, visible as a disruption of the regular hexagonal packing, as the surviving cells appear to migrate and/or expand to maintain the integrity of the epithelial layer (11, 14, 15). An example of this can be seen in a flat mount from a P42 *rd1* mouse, in which the regular polygonal geometry of the RPE is replaced with distorted cells of various shapes (Figure 1B).

To test whether overexpression of Nrf2 specifically in the RPE would protect it from the stress of retinal degeneration, an AAV vector that utilizes the human Best1 promoter (16) to selectively express genes only in the RPE was used. RPE flat mounts from a P42 *rd1* mouse that received subretinal injections of AAV-Best1-Nrf2 at P1 in the right eye and a control virus (AAV-hRedO-H2b-GFP) in the left eye were analyzed. The control virus used the human red opsin promoter (hRedO), which is not expressed in the RPE, to drive expression of GFP. There was significant rescue of the regular hexagonal geometry in the RPE layer in the eye receiving AAV-Best1-Nrf2 (Figure 1C), which included a suppression of the loss of RPE cells.

To determine whether overexpression of Nrf2 in the retina leads to a similar preservation of the RPE, we used the hRedO promoter to drive Nrf2 overexpression in the photoreceptor layer. In eyes of P42 *rd1* mice that received P1 subretinal injections of AAV-hRedO-Nrf2, the hexagonal array of the RPE monolayer appeared as disrupted as the control eye injected with AAV-hRedO-H2b-GFP (Figure 1, D and E). Thus, direct overexpression of Nrf2 in the RPE is necessary to preserve its morphology when the adjacent retina degenerates in *rd1* mice.

*Nrf2expressionintheRPEisnecessaryandsufficienttoreducethedisturbanceinthedistributionofrd1conephotoreceptors*. Our previous work showed that an AAV using the CMV promoter to drive robust expression of Nrf2 in cones and the RPE significantly prolonged the survival and function of cone photoreceptors (7). To determine if expression only in the RPE might also benefit cones, retinal flat mounts from eyes that received subretinal coinjections of AAV-Best1-Nrf2 and AAV-hRedO-H2b-GFP in one eye and AAV-hRedO-H2b-GFP in the corresponding eye were examined for their cone number and distribution. The H2b tag results in accumulation of GFP in nuclei, making it straightforward to identify and quantify cones, the only photoreceptors that survive at these time points in untreated *rd1* mice (17). We found that this was a less ambiguous way to count cones in degenerating retinas than was IHC against antigens such as cone opsin and arrestin. We and others have found that rods in the *rd1* mouse do not survive, despite AAV-CMV-hNrf2 (WX and CLC, unpublished observations) or treatment with a systemic antioxidant (18); thus, hRedO-H2b-GFP served as a reliable marker for cones only.

**Figure 2:**
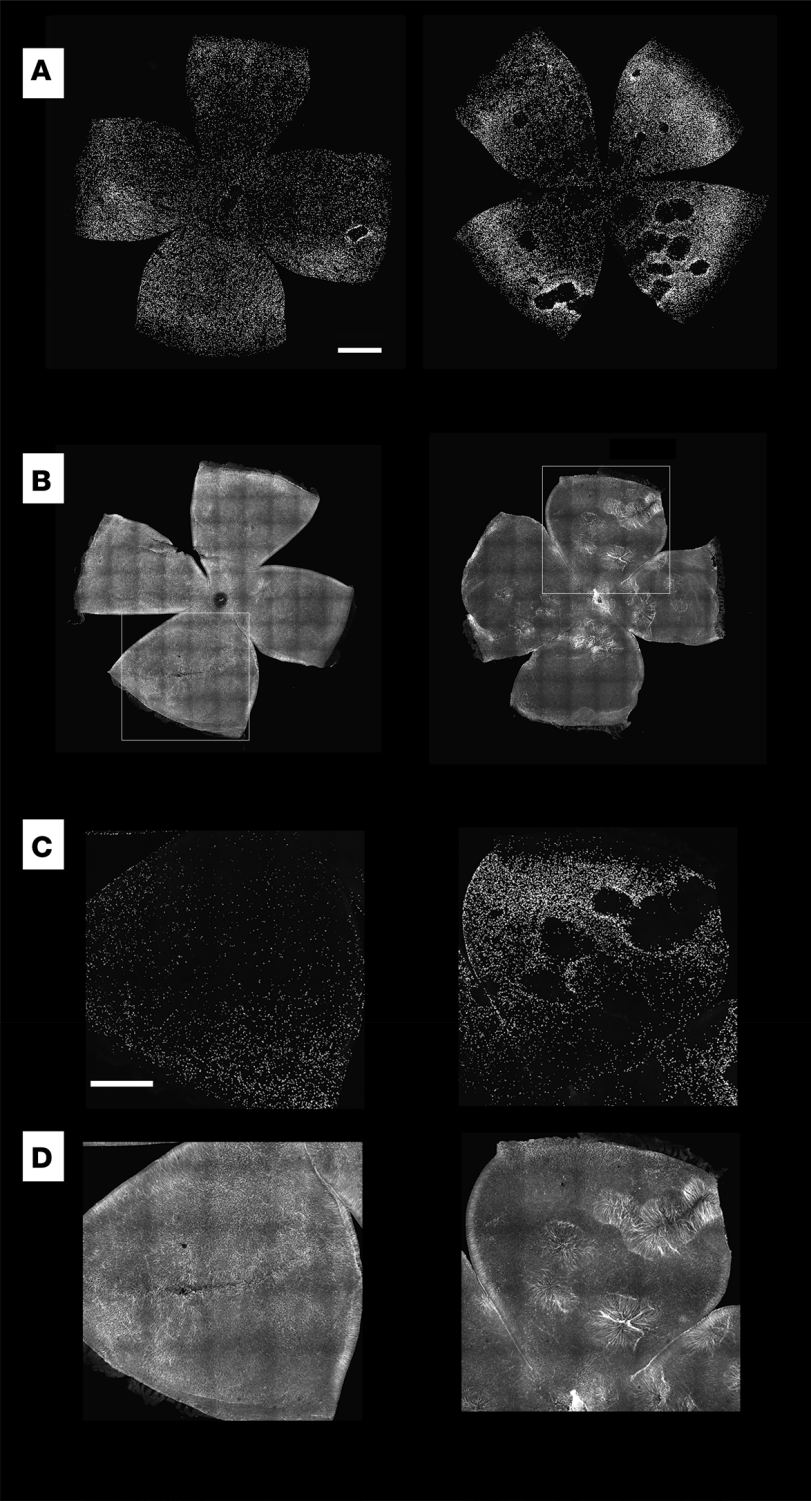
Changes in retinal structure in *rd1* mice and the effect of overexpression of Nrf2 in the RPE. (**A**) Confocal images of pairs of retina flat mounts from the right and left eyes of a P41 *rd1* mice that received a coinjection of AAV-Best1-Nrf2 and AAV-hRedO-H2b-GFP in the left eye (left panel) and control AAV-hRedO-H2b-GFP in the right eye (right panel) (*n* = 13 mice). Each point of fluorescence is the nucleus of a cone. Note the disruptions, which appear as “holes” or “craters” in the cone mosaic, particularly in the right eyes. (**B**) Confocal images of a pair of retinas from the right and left eyes of P48 *rd1* mouse, with anti-GFAP labeling, demonstrating fairly uniform GFAP distribution in the left retina from the eye that received AAV-Best1-Nrf2 but significant upregulation and distortion of the radial Muller glial fibers in areas of cone loss in the right retina from the eye that received AAV-hRedO-H2b-GFP only (*n* = 2 mice). Insets show higher magnification of these areas with GFP (**C**) and GFAP (**D**). Scale bars: 500 μm for **A** and **B**, 200 μm for **C** and **D**.

**Figure 3:**
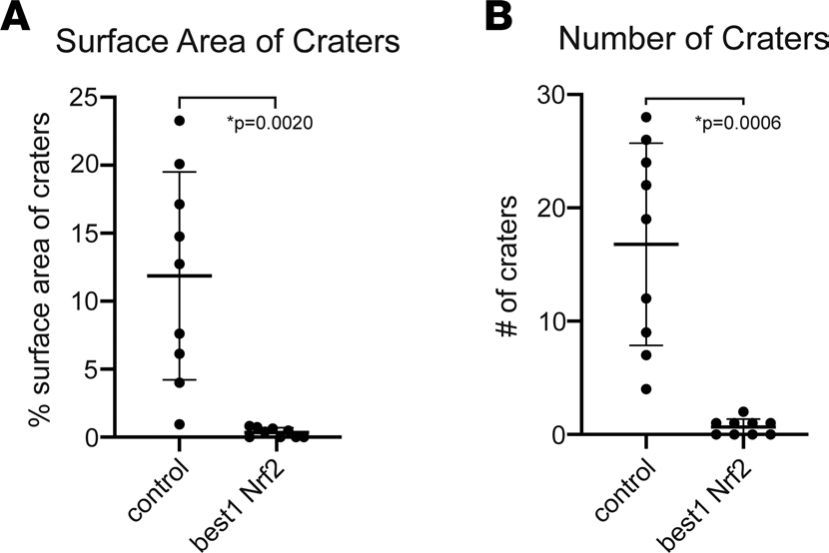
Quantitative analysis of the arrangement of cones in retinas in the presence and absence of Nrf2 overexpression in the RPE. (**A**) Plot showing the surface area of craters as a percentage of total surface area in retinal flat mounts treated with AAV-Best1-Nrf2 and AAV-hRedO-GFP-H2b compared with control (AAV-hRedO-GFP-H2b only) in P40-P60 mice. Area was measured at the outer, apical retinal surface (*n* = 9 mice). (**B**) Plot showing the number of craters counted in each retina flat mount from the same conditions (*n* = 9 mice). Data shown as mean ± SD. *P* < 0.005 by paired *t* test.

Cones are usually fairly evenly distributed across the retina. In *rd1* mice, there is a loss of cone photoreceptors in circumscribed domains, appearing to form craters in the outer nuclear layer when viewed as a retinal flat mount (Figure 2A). The radial fibers of Müller glia can be seen spanning these craters in areas devoid of cones (Figure 2, B–D). Eyes that received subretinal coinjections of AAV-Best1-Nrf2 and AAV-hRedO-H2b-GFP in one eye at P1 had significantly less cratering measured by both fewer numbers of craters and less surface area covered by craters than their counterpart eyes infected with AAV-hRedO-H2b-GFP (Figure 3, A and B). We also quantified the number of cones rescued as a ratio of central GFP^+^ nuclei to total GFP^+^ nuclei. Because our counting methodology is dependent on infection efficiency of AAV hRedO-H2b-GFP, and because significant cone degeneration occurs centrally, this allowed us to normalize for variations in viral infection efficiency across different eyes. There was a statistically significant, but small, improvement in this ratio for eyes infected by AAV-Best1-Nrf2 in comparison with control (Supplemental Figure 1; supplemental material available online with this article; https://doi.org/10.1172/jci.insight.145029DS1). Thus, we conclude that, in addition to preserving the morphology of the RPE, overexpression of Nrf2 in the RPE also reduces the disruption to the regular arrangement of cones.

**Figure 4:**
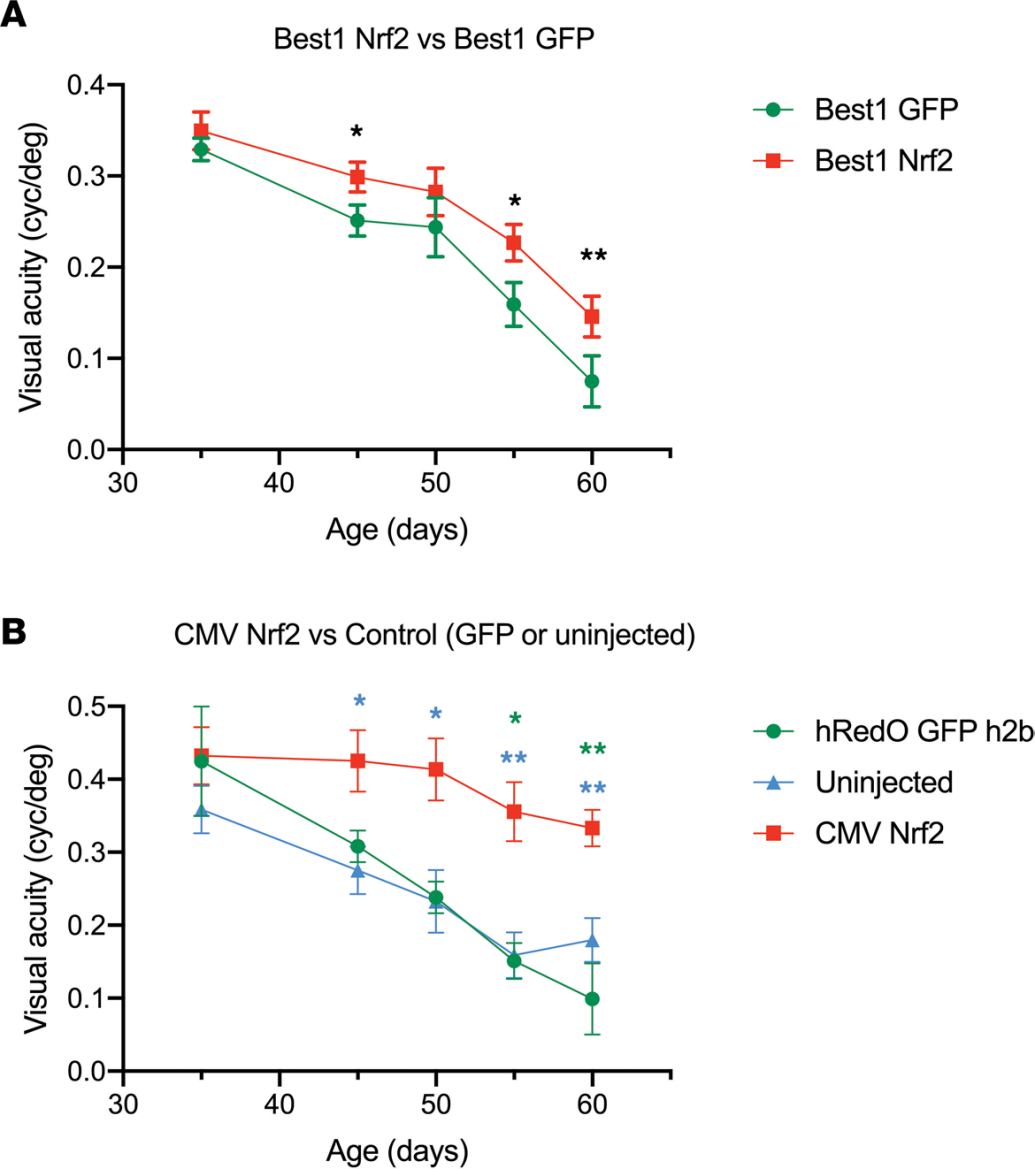
Visual acuity of *rd1* mice with and without AAV-Best1-Nrf2. (**A**) Visual acuity (measured by the optomotor assay) for *rd10* mice that were injected in one eye with AAV-Best1-Nrf2 and AAV-Best1-GFP and the other eye with AAV-Best1-GFP only (*n* = 19 mice for all time points except p50 [where only 9 of the mice were tested] and P60 [where *n* = 16 because 2 of the mice died between P55 and P60 and 1 did not cooperate with the test]). Data shown as mean ± SEM. *P<0.05 and **P<0.005 by paired *t* test. (**B**). Optomotor visual acuity for *rd10* mice that were injected in one eye with AAV-CMV-Nrf2 plus AAV-hRedO-GFP-H2b and in the other eye with AAV-hRedO-GFP-H2b or no injection. In *rd10* mice, eyes injected with AAV-hRedO-GFP-H2b and uninjected eyes were shown to have no difference in visual acuity between P45 and P60 in previously published work; therefore, both were used as controls (81) (*n* = 14 mice; one eye injected with AAV-CMV-Nrf2 and the contralateral eye injected with AAV-hRedO-GFP h2b [*n* = 3] or uninjected [*n* = 11] for all time points except P60, where *n* = 13 mice because 1 mouse developed a cataract and was excluded). Data shown as mean ± SEM. *P<0.05 and **P<0.005 by 1-way ANOVA (green asterisk indicates AAV-CMV-Nrf2 versus AAV-hRedO-GFP-H2b comparison, and blue asterisk indicates AAV-CMV-Nrf2 versus uninjected comparison).

*Nrf2expressionintheRPEleadstoamodestrescueofvision*. The *rd10* mice also have a mutation in *Pde6b*, which is only a partial loss of function, compared with the *rd1* allele, which is a total loss of Pde6b function. The *rd10* strain retains enough vision for an assessment to be made using the optomotor test for effects of a treatment on acuity. In the eyes of *rd10* mice treated with Best1-Nrf2, there was a small but statistically significant rescue of vision compared with the paired control eye (Figure 4A). This effect was significantly lower than in eyes that received CMV-Nrf2, which results in overexpression of Nrf2 in both photoreceptors and RPE (Figure 4B). As our previous work showed that CMV-Nrf2 promotes survival of a significantly greater number of cone photoreceptors, it is not a surprise that AAV-CMV-Nrf2 gives a much more marked rescue on the optomotor assay (7).

### Nrf2 overexpression in the RPE stabilizes the interface with photoreceptors.

The RPE and photoreceptors are closely associated in the subretinal space, with the apical microvilli of the RPE interdigitating with the outer segments of the photoreceptors (19).

**Figure 5:**
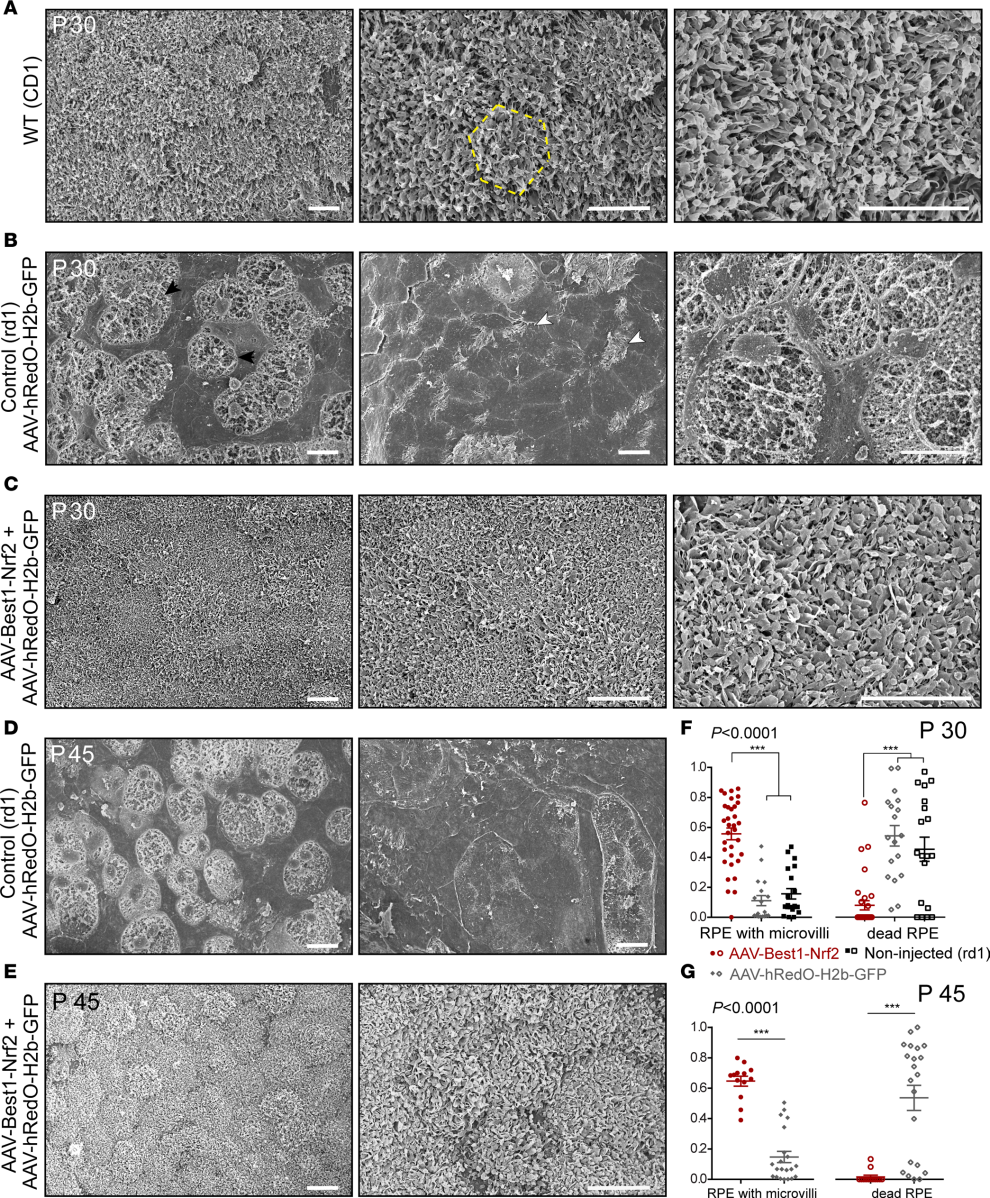
Scanning EM analysis of RPE from WT, *rd1*, and *rd1* mice rescued with AAV-Best1-Nrf2. (**A**) Representative scanning EM photomicrographs of P30 RPE flat mounts from a WT (CD1) mice (*n* = 3). RPE cells were uniform in size and shape and formed a hexagonal array of cells within the flat mount (left panel). The yellow hexagonal outline designates a single cell (middle panel). Extensive long processes formed a dense carpet of microvilli across the entire apical surface of the RPE (right panel). (**B**) Representative scanning EM photomicrographs of a P30 RPE flat mount from an *rd1* retina injected with AAV-hRedO-H2b-GFP (*n* = 5). Cells were highly variable in size and shape (left panel). Large cells exhibited no apical processes with smooth apical surfaces, and cells had short apical processes (middle panel, white arrowheads). There were large areas of deterioration noted in the entire flat mount where the cells appear to have died, as indicated by cellular debris (left panel, black arrowheads; middle panel, white arrowheads). (**C**) Representative scanning EM photomicrographs of P30 RPE flat mount from retinas *rd1* coinjected with AAV-Best1-Nrf2 and AAV-hRedO-H2b-GFP (*n* = 3). Uniform geometry of polygonal RPE with dense carpet of intact microvilli. (**D**) Representative scanning EM photomicrographs of P45 RPE flat mount from *rd1* eyes injected with AAV-hRedO-H2b-GFP (*n* = 3). Cells were highly variable in size and shape, and there were large areas of cellular debris (left panel). Cells exhibited no apical processes with smooth apical surfaces (right panel). (**E**) Representative scanning EM photomicrographs of P45 RPE flat mount from retinas *rd1* coinjected with AAV-Best1-Nrf2 and AAV-hRedO-H2b-GFP (*n* = 3). Polygonal RPE with well-preserved microvilli remained intact at P45. Scale bars: 10 μm. (**F**) Quantification of fraction of RPE surface area at P30 with well-preserved microvilli and areas of RPE with no apparent live RPE cells, injected with AAV-Best1-Nrf2 and AAV-hRedO-H2b-GFP at P1 or noninjected (*n* = 3–5 mice/group). (**G**) Quantification of fraction of RPE surface area at P45 with well-preserved microvilli and areas of RPE with no apparent live RPE cells, injected with AAV-Best1-Nrf2 and AAV-hRedO-H2b-GFP at P1 (*n* = 3 mice/group). Data shown as mean ± SEM. ***P<0.0001 by 1-way ANOVA.

In order to evaluate the morphology of the RPE at high resolution, electron microscopy (EM) was used. The ultrastructure of the apical surface of the RPE was first analyzed by scanning EM. In WT mice at P30, most of the RPE cells were uniform in size and shape and were arranged in a regular hexagonal array (Figure 5A). Numerous, thin processes protruded from the apical surface, forming a dense carpet of microvilli across the entire apical surface of the RPE (Figure 5A). Examination of eyes of untreated *rd1* mice (data not shown), or eyes of mice injected with AAV-hRedO-H2b-GFP (Figure 5B), showed strikingly aberrant RPE cellular morphology. RPE cells were highly variable in terms of size and shape. They could be enlarged, elongated, spindle-shaped, small, and/or rounded. Very large cells had no apical processes, whereas some smaller cells showed a few apical processes (Figure 5B, white arrowheads), along their epithelial cell borders only. In some cases, there were large areas where the cells most likely had died, as no RPE cellular structure could be discerned, and there appeared to be cellular debris (Figure 5B, black arrowheads). In contrast, the contralateral eyes of the *rd1* mice that received an injection with AAV-Best1-Nrf2 plus AAV-hRedO-H2b-GFP showed hexagonal, uniform cells with significant preservation of microvilli on their apical surface at P30 (Figure 5C). Later time points, P45 and P60, showed similar results in retinas infected with AAV-Best1-Nrf2 plus AAV-hRedO-H2b-GFP (Figure 5, D–E, and data not shown).

To quantitatively evaluate the relative surface areas of RPE cells with well-preserved apical microvilli, versus no apical processes, as well as areas of presumed RPE death, we analyzed RPE samples from P30 using scanning EM photomicrographs and ImageJ software. Comparisons were made between those eyes that received AAV-Best1-Nrf2 plus AAV-hRedO-H2b-GFP versus those injected with only AAV-hRedO-H2b-GFP. Those with Best1-Nrf2 showed significantly higher relative surface areas of RPE cells with well-preserved microvilli and lower surface areas with dead cells compared with noninjected *rd1* or those injected with AAV-hRedO-H2b-GFP (Figure 5F). The relative surface areas of RPE cells in retinas infected with AAV-Best1-Nrf2 and AAV-hRedO-H2b-GFP, versus AAV-hRedO-H2b-GFP only, also were evaluated at a later time point, P45 (Figure 5G). Significant preservation of RPE with microvilli was also seen at this time point when AAV-Best1-Nrf2 was delivered.

To exclude the potential loss of microvilli from the RPE during scanning EM sample preparation, which requires separation of the retina and RPE, and to evaluate additional aspects of ultrastructure, transmission EM (TEM) was performed on P30 and P60 eyes from AAV-Best1-Nrf2 treatment.

**Figure 6:**
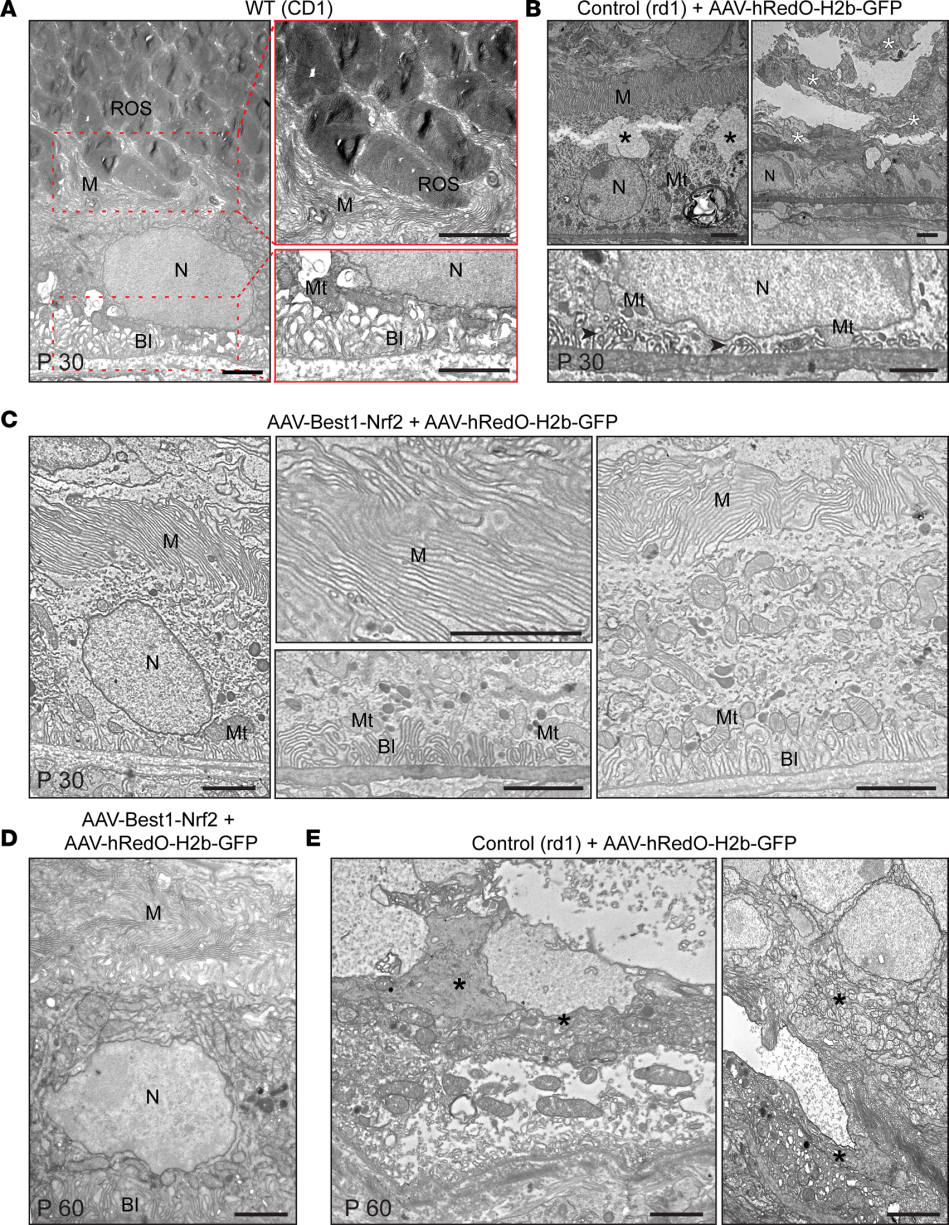
Transmission electron microscopic analysis of RPE and photoreceptor interface in treated and untreated eyes. (**A**) Representative TEM photomicrographs of P30 RPE from a WT retina (left panel) (*n* = 3). Carpet-like microvilli formed clusters or single villi oriented toward and between ROS at the apical RPE (right top panel). Infoldings of the basal membrane can be seen in the (left bottom panel). (**B**) Representative TEM photomicrographs of P30 RPE from an *rd1* mouse injected at P1 with AAV-hRedO-H2b-GFP (*n* = 4). Degenerative changes in the RPE can be seen, including vacuolization in the cytoplasm in the apical region (left top panel, black asterisks), a reduced number of shortened infoldings (black arrowheads) and mitochondria in the basal region (bottom panel), and areas containing cellular debris and dead RPE cells (right top panel, white asterisks). (**C**) Representative TEM photomicrographs of P30 RPE from an *rd1* mouse injected at P1 with AAV-Best1-Nrf2 and AAV-hRedO-H2b-GFP (*n* = 3). Microvilli appeared to form a compact barrier between the RPE and inner retina (left and right panel). The basal infolding region had parallel and long microvilli surrounded by mitochondria (middle bottom panel). (**D**) Representative TEM photomicrographs of P60 RPE from an *rd1* mouse injected at P1 with AAV-Best1-Nrf2 and AAV-hRedO-H2b-GFP (*n* = 3). (**E**) Representative TEM photomicrographs of P60 RPE from an *rd1* mouse injected at P1 with AAV-hRedO-H2b-GFP shows RPE death and cellular debris (black asterisks) (*n* = 3). BI, basal infoldings; M, microvilli; Mt, mitochondria; N, nucleus; ROS, rod outer segment. Scale bars: 2 μm.

In a cross section, it is possible to distinguish 3 regions of an RPE cell: a region with basal infoldings, the main cytoplasmic compartment of the cell (including the basal and apical cytoplasmic zones), and microvilli. At P30 in nondegenerated WT mice, the region of microvilli was composed of narrow, elongated cytoplasmic processes in clusters, or single processes oriented toward and between rod outer segments (ROS; Figure 6A). The RPE cytoplasmic compartment contained a variety of organelles. The basal region featured long, narrow infoldings, mostly parallel with each other, and parallel to the microvilli (Figure 6A). The basal region also included mitochondria. In *rd1* eyes injected with AAV-hRedO-H2b-GFP, degenerative changes in the RPE were observed using TEM (Figure 6B). These included cytoplasm vacuolization in the apical region, severe disorganization of microvilli (reduced in number of shortened infoldings and mitochondria in the basal region), and areas containing cellular debris that appeared to be dead RPE cells (Figure 6B). In contrast, with AAV-Best1-Nrf2 plus AAV-hRedO-H2b-GFP, the slender microvilli between the RPE and retina were well preserved and resembled those in CD1 eyes (Figure 6C). They appeared to form a compact barrier between photoreceptors and the RPE. The basal infolding region of the RPE had parallel, long microvilli surrounded by mitochondria of various shapes and sizes. The differences between AAV-Best1-Nrf2–injected eyes and those of controls persisted at P60 (Figure 6, D and E).

*TranscriptomechangesduetoNrf2overexpressionintheRPE*. Oxidative stress has been shown to increase after the death of rods, and it is thought to be one of the mechanisms that lead to cone death (9). Nrf2 is best known for its role as a master regulator of antioxidant genes, although it also affects the transcription of genes involved in a wide range of other processes related to the stress response (20). In order to better understand the mechanism by which Nrf2 protects the RPE, we performed transcriptome profiling of RPE from *rd1* mice at 2 different ages with or without overexpression of Nrf2. The levels of degeneration can vary, even between *rd1* mice of the same age and litter; to reduce variability, we structured our comparisons so that one eye of each mouse received AAV-Best1-Nrf2 and the other eye received a control virus (AAV-Best1-GFP). We also performed transcriptome profiling of RPE from WT (CD1) mice with or without overexpression of Nrf2 to evaluate changes associated with Nrf2 overexpression relative to those changes seen in a degenerating eye.

**Figure 7:**
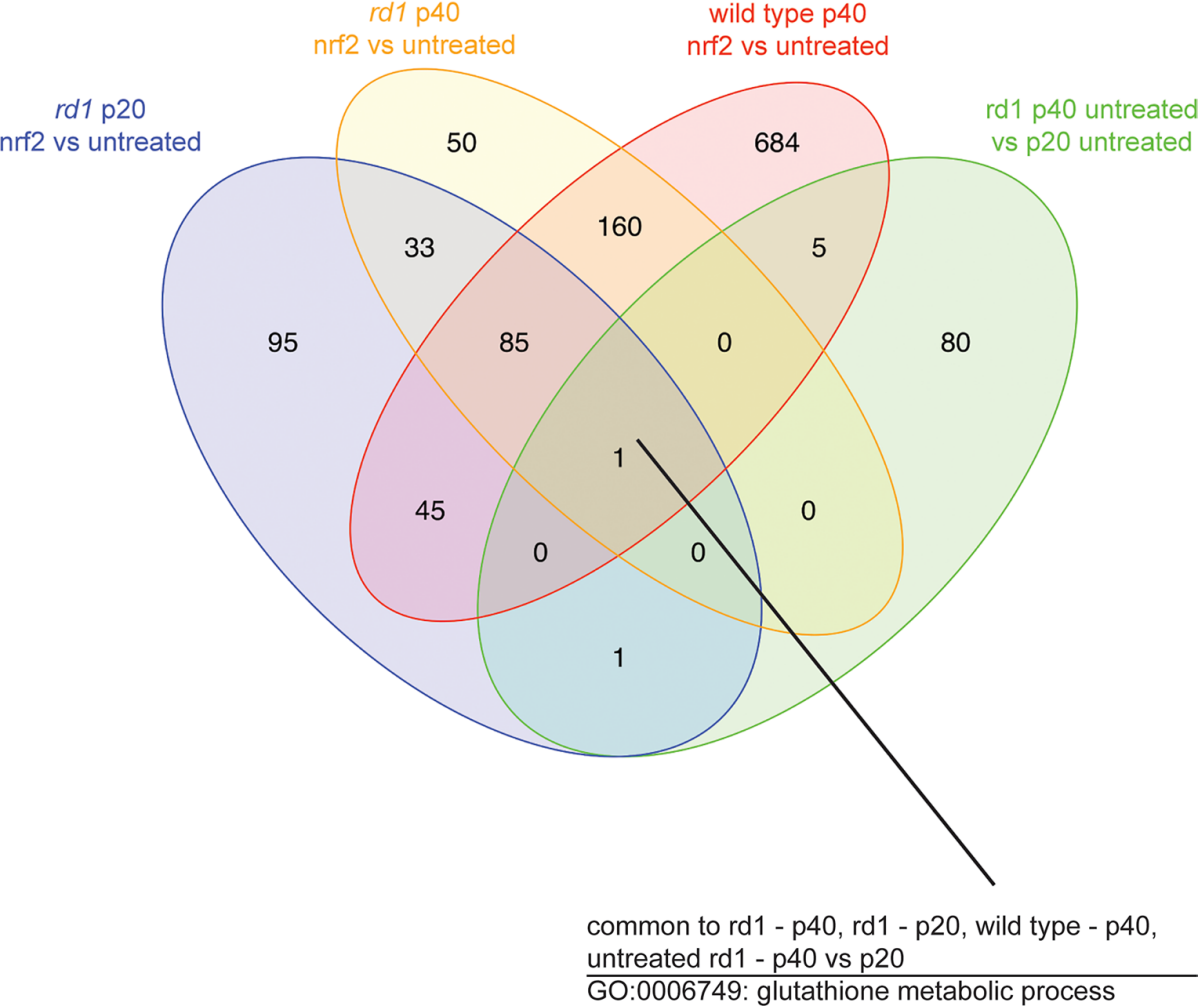
Transcriptional profiling of RPE from WT and *rd1* mice with or without overexpression of Nrf2. Venn diagram showing the number of gene ontology (GO) terms for biologic processes overrepresented in each comparison and the number of biological processes shared across the different comparisons. The number within each overlapped area denotes the number of shared biological processes between each overlapped comparison. A single GO term, glutathione metabolism, was overrepresented in all comparisons (all 3 comparisons that involved Nrf2 overexpression, as well as untreated P20 versus P40 *rd1* mice) (*n* = 6 mice for p20, 4 mice for p40 rd1, and 5 mice for p40 WT)

Overall, we detected 2223 differentially expressed genes between treated and untreated eyes from *rd1* RPE at P20, 1640 differentially expressed genes between treated and untreated *rd1* RPE at P40, and 1807 differentially expressed genes between treated and untreated WT RPE at P40. Nrf2 had wide-ranging effects on the transcriptome of RPE cells in WT mice, ranging from an increase in oxidative defense and detoxification gene expression to changes in the expression of genes related to the immune response and metabolism (Supplemental Table 1). To better understand which effects may be critical to the rescue of the RPE, we performed a combinatorial analysis of the differentially expressed genes from the different comparisons. We hypothesized that the most direct Nrf2 effect on rescue would be one in which Nrf2 induced a change in one direction and the natural history of the degeneration induced a change in the opposite direction. There was a single biological process — glutathione metabolic process (GO:0006749; http://www.informatics.jax.org/go/term/GO:0006749) — common to all 4 of these comparisons (Figure 7). Glutathione is the major antioxidant as well as an important participant in many cellular processes (21). Among several genes related to glutathione metabolism upregulated by Nrf2 were *Gclc* (glutamate cysteine ligase catalytic subunit ligase), and *Gclm* (glutamate cysteine ligase modifier subunit), which encode the catalytic and regulatory subunits, respectively, of glutamatecysteine ligase (GCL), the first and rate-limiting enzyme in the glutathione synthesis pathway (22). *Gss*, the gene for glutathione synthetase (Kyoto Encyclopedia of Genes and Genomes [KEGG] 6.3.2.3; https://www.genome.jp/dbget-bin/www_bget?ec:6.3.2.3), the second enzyme in the glutathione synthesis pathway — as well as γ-glutamyl transpeptidase (KEGG 2.3.2.2), which strips the cysteine off of extracellular GSH to facilitate intracellular synthesis of GSH — also had increased expression following Nrf2 introduction at p20 in *rd1* and at p40 in *rd1* and WT; they were downregulated in untreated rd1 RPE from p20 to p40 (23). *Slc7a11*, which encodes for the xCT subunit of the cysteine transporter that can increase the availability of this amino acid for glutathione synthesis (Supplemental Figure 2), also was upregulated from Nrf2 overexpression and downregulated in untreated *rd1* RPE from P20 to P40. *Gsr,* the gene for glutathione reductase (KEGG 1.8.1.7), which catalyzes the reduction of the glutathione disulfide to the protective sulfhydryl form, was upregulated by Nrf2 and unchanged in the untreated *rd1* RPE from p20 to p40. Some genes, such as those for glutathione reductase and glutathione peroxidase isozymes, that would be expected to be involved in a stress response, were upregulated in Nrf2-treated tissue, as well as untreated rd1 tissue. These data suggest that there is some endogenous attempt by the RPE to defend against the stress from degeneration of the neighboring retina and that Nrf2 can augment this response.

**Figure 8:**
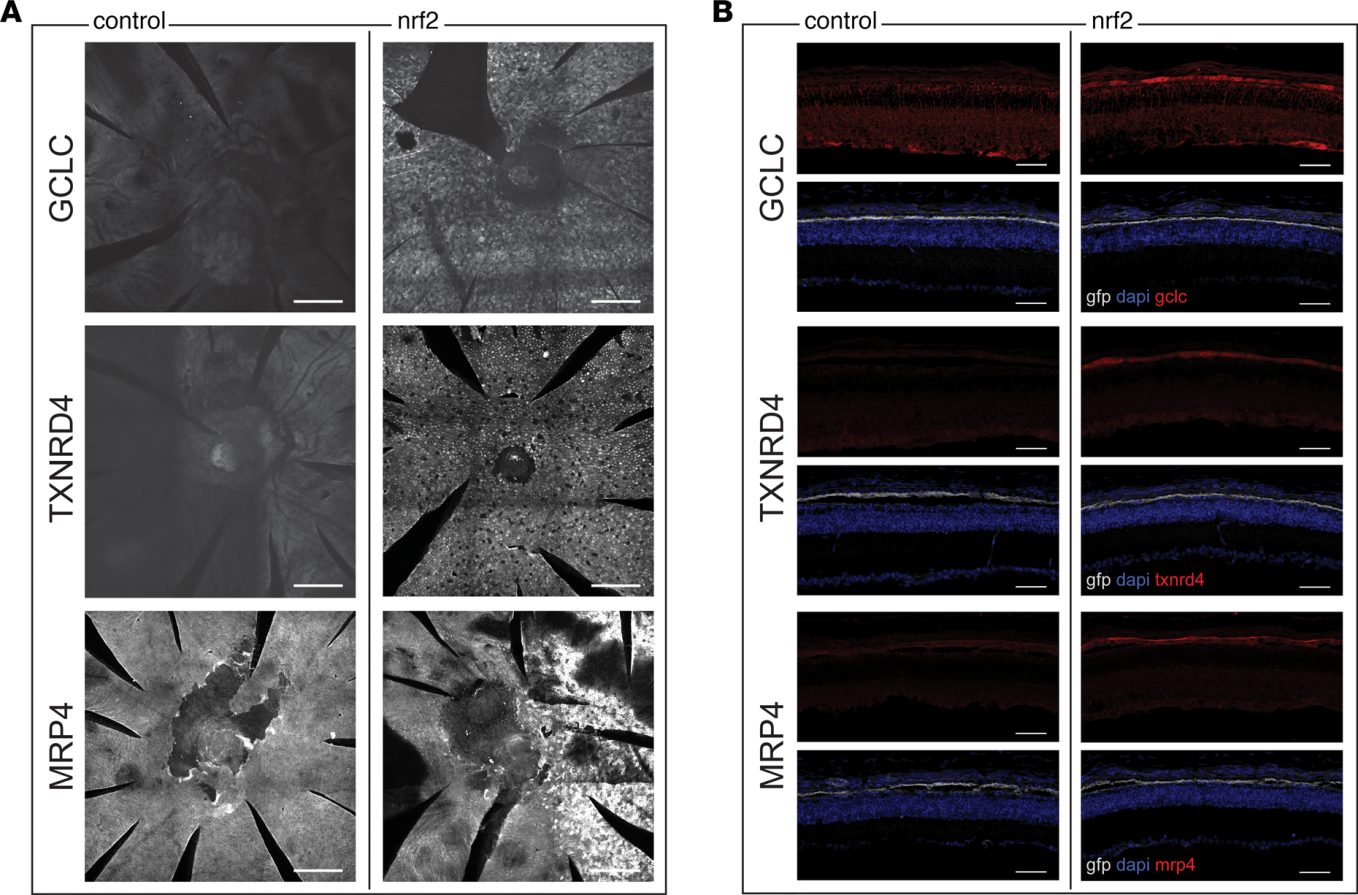
IHC for DE genes from transcriptional profiling. (**A**) RPE flat mounts showing staining for antibodies against GCLC, TXNRD1, or MRP4. Each row shows flat mounts from pairs of left and right eyes from a P40 *rd1* mouse (1 pair per antibody). In the left eyes, only a control AAV-hRedO-GFP was injected. In the right eyes, the eye was also injected with AAV-Best1-Nrf2. Staining was absent in the eyes on the left-hand side, which did not have overexpression of Nrf2. In the pair of eyes stained with MRP4, only the right half of the eye was infected with Nrf2. Scale bars: 200 μm. (**B**) Sections from pairs of left and right eyes from P40 *rd1* mice. In the left eyes, only a control virus, AAV-Best1-GFP was injected. In the right eye, the eye was also injected with AAV-Best1-Nrf2. Selective expression of GFP showing the RPE layer was seen in both eyes (*n* = 2 per antibody). Scale bars: 50 μm.

Antibody staining for GCLC was carried out and showed substantially more signal in RPE flat mounts and sections from eyes with overexpressed Nrf2 relative to control tissue (Figure 8, A and B). Thioredoxins are another major antioxidant system in mammals regulated by Nrf2. The product of thioredoxin reductase 1 (*Txnrd1*) is the major cytosolic reducer of thioredoxins in mice (24, 25). Expression of *Txnrd1* also was upregulated by Nrf2 in the RPE from P20 and P40 *rd1* mice, as well as P40 WT mice, relative to untreated WT or *rd1* mice. Antibody staining for TXNRD1 in RPE flat mounts and sections showed increased expression in eyes with Nrf2 overexpression, consistent with the transcriptome profiling results (Figure 8). Finally, we stained for the product of *Abcc4* (Figure 8)*,* which was upregulated in the RPE of P20 *rd1* mice, p40 *rd1* mice, and p40 WT mice treated with AAV-Best1-Nrf2, relative to untreated WT or *rd1* mice. *Abcc4* encodes multidrug resistance protein 4 (MRP4), which is a member of the superfamily of ATP-binding cassette (ABC) transporters. MRP4 has been shown to be induced by Nrf2 and facilitates glutathione efflux, which has been noted to occur in cultured RPE as well as other systems, where it may be a potential defense mechanism against oxidative stress (26–29). Staining for this protein also showed greater signal in the Nrf2-treated eyes.

## Discussion

It has long been known that the RPE is involved in the phagocytosis and maintenance of the photoreceptor outer segments and the recycling of the photopigment of the visual cycle (30, 31). In addition, there is a growing appreciation of a metabolic interplay between the RPE and the photoreceptors (32, 33). It is, thus, not a surprise that retinal degeneration, even one secondary to a rod-specific mutation, would lead to disturbances in the RPE. Fundus imaging of mice with *rd1* mutations has identified RPE depigmentation after photoreceptor loss (34). In patients with retinitis pigmentosa, hypopigmentation can be seen on direct examination, as well as upon examination of autofluorescence (35). Following photoreceptor loss, RPE cells can migrate out of the epithelial monolayer into the inner retina (10).

Previous histological studies in *rd1* mice have shown that RPE microvilli are initially elongated during the first 3 postnatal weeks when there is significant photoreceptor degeneration (36). Between P28 and P42, there is thinning of the RPE with loss of apical microvilli, basal infoldings, and mitochondria. By P56, in areas where there is photoreceptor loss and glial hypertrophy, there is concomitant RPE loss. More recently, the disruption of the cytoskeletal architecture of the RPE in mice with *Pde6b* mutations has been described in elegant quantitative analyses of RPE morphology by Nickerson and colleagues (11, 15). However, most of the investigation of RPE in retinal disease has actually been in the context of age-related macular degeneration (AMD). Nonetheless, as the RPE cytoskeletal changes reported in this work, as well as by Nickerson and colleagues (11, 15), have also been described in AMD, there are likely aspects of RPE biology discovered through AMD research that are relevant across both disease types (37).

In AMD, there has been a focus on the RPE as the potential origin of the disease, as well as attention on the role of oxidative stress in RPE dysfunction (38). The RPE is sandwiched between the highly oxygenated blood of the choroid, and the retina, which is constantly under photooxidative stress. Adding to this stress, the photoreceptors shed lipid-rich outer segments into the subretinal interface with the RPE, which are phagocytosed by the RPE (39–41). Since the RPE is normally functioning in a demanding environment, additional stress due to disease likely overcomes the endogenous defense mechanisms of the RPE and causes degeneration. In AMD, the 2 most important risk factors are aging and smoking, which both lead to additional oxidative stress (42). The RPE cells are full of mitochondria to assist in their maintenance and to generate reactive oxygen species (ROS) for phagocytosis (43). As the mitochondria age, the prolonged exposure to ROS leads to the accumulation of mitochondrial DNA (mtDNA) mutations, which decrease mitochondrial redox function, further increasing ROS production and mtDNA damage (1, 44, 45). Smoking can further introduce ROS with the many oxidative components in cigarette smoke (46).

For retinitis pigmentosa, the origin of pathologic oxidative stress is different than in AMD. As rods are the major inhabitants of the outer nuclear layer, and since they consume high amounts of oxygen, their loss in retinitis pigmentosa leads to hyperoxic conditions (4). The resulting oxidative stress is a favored hypothesis for death of bystander cells in retinitis pigmentosa. In support of this being a causal relationship (5, 47, 48), multiple groups have shown that systemic administration of antioxidants prolongs cone survival in *rd1* and *rd10* mice (9, 18, 49). Our own previous investigation showed that levels of hydroethidine and acrolein staining were lower in AAV-CMV-Nrf2–treated mouse models of retinitis pigmentosa (7). Thus, it is likely that lessons learned through the AMD literature concerning the mechanisms used by the RPE to handle oxidative stress may be relevant in retinitis pigmentosa.

AMD investigators have extensively studied ways by which the RPE protects itself from oxidative stress. One of the most significant is the Nrf2 transcription pathway. Nrf2-KO mice, in the last third of their lifespan, begin to show age-related accumulation of drusen-like deposits, inflammatory proteins, and autophagy-related vacuoles in the RPE and outer retina (50). These are reminiscent of AMD in humans (51, 52). Nrf2 signaling is less robust in aged mice than in young mice, and it is further impaired by cigarette smoking, paralleling the leading risk factors for AMD (53, 54). One of the pathways regulated by Nrf2 is the oxidative damage defense pathway. Acutely increasing the RNAs for catalase and SOD, as well as more chronically increasing glutathione and thioredoxin levels, are 2 of the ways in which Nrf2 helps a cell fight oxidative damage (55). Our transcriptome results are in line with the importance of oxidative stress, as the single process that was both increased by Nrf2 and decreased in untreated RPE was related to the major antioxidant pathway of glutathione. Our transcriptome profiling also showed significant upregulation of other oxidative defense pathways (e.g. catalase, SOD, and thioredoxin), suggesting that rescue by overexpression of Nrf2 in the RPE of the retinitis pigmentosa mouse model works, at least in part, through reduction of oxidative stress. Our previous work delivering SOD2 and catalase using AAV (7), as well as overexpression of *Gpx4* in a transgenic mouse model by Lu and colleagues (56), showed that they, too, were beneficial, though not as potent as Nrf2 overexpression (7). Although beyond the scope of this paper, we also found many additional pathways unrelated to antioxidant defense that were modulated by Nrf2 overexpression. This is not a surprise, as Nrf2 is well known to modulate many pathways related to inflammation and metabolism (57, 58). As the interplay of oxidative stress, inflammation, and metabolic change has been the recent focus of hypotheses concerning the cause of AMD, such changes may work alongside the antioxidant systems to effect a greater rescue (33, 59). As an example, consistent with studies in cancer cells, we found that Nrf2 significantly boosted several genes in the pentose phosphate pathway, including those for glucose-6-phosphate dehydrogenase (*G6pd*), transaldolase 1 (*Taldo1*), and phosphogluconate dehydrogenase (*Pgd*) (60). A recent study of RPE metabolism found that the pentose phosphate pathway is of very high importance in the RPE/choroid relative to the retina (61). Nrf2’s ability to enhance throughput through a prominent pathway of the RPE, in addition to its more well-known effects on oxidative stress, suggests that it may provide additional benefits beyond those provided by an antioxidant.

In conclusion, we show here that, in 2 *pde6b* mouse models of retinitis pigmentosa, the RPE shows significant signs of stress after the initial loss of rods. Selective overexpression of Nrf2 in the RPE with an AAV construct driven by the Best1 promoter is sufficient to produce a robust rescue effect. There is also a significant reduction in the disruptions seen in the cone photoreceptor mosaic, as well as a modest slowing of vision loss. Transcriptome profiling showed Nrf2-mediated upregulation of antioxidant pathways and disturbances in these pathways in RPE from control mice. These are the same pathways that are implicated in the pathogenesis of AMD. Given that Nrf2 overexpression modulates these pathways and reverses the degeneration of RPE seen in RP and AMD, our results suggest that Nrf2 overexpression may be a good candidate to slow the pathogenesis of nonexudative AMD, as well as retinitis pigmentosa.

## Methods

### Animals.

CD-1 (catalog 022) and *rd1* (FVB/N, catalog 207) mice were purchased from Charles River Laboratories. *Rd10* mice (catalog 004297) were purchased from The Jackson Laboratory. Animals were bred and maintained at Harvard Medical School on a 12-hour alternating light and dark cycle.

### Plasmids.

A vector utilizing the human Best1 promoter was created by replacing the CMV promoter of the AAV-CMV-PI-EGFP-WPRE-bGH plasmid, a gift from James M. Wilson, University of Pennsylvania (Philadelphia, Pennsylvania, USA) with the –540/+38 bp region of the human Best1 promoter (16). AAV-Best1-Nrf2 was cloned by replacing the EGFP coding sequence with the GCCGCCACC Kozack sequence, followed by the human cDNA clone of Nrf2 (NM_006164.4) obtained from Thermo Fisher Scientific. An AAV vector combining the hRedO promoter with the posttranscriptional regulatory element WPRE–bGH polyA was a gift from Botond Roska (Friedrich Miescher Institute for Biomedical Research, Basel, Switzerland) using the promoter region originally developed by Wang et al. (62, 63). AAV-hRedO-Nrf2 was cloned by replacing the GFP coding sequence with the aforementioned human cDNA clone of Nrf2. AAV-hRedO-H2b-GFP was cloned by adding an H2b nuclear localization sequence to the original vector (64). AAV-CMV-Nrf2 was cloned by taking the aforementioned human cDNA clone of Nrf2 and inserting this into the AAV-MCS8 empty vector (Harvard Medical School DF/HCC DNA Resource Core) with Not1/XhoI sites. Capsid serotypes used were AAV8 for all vectors except AAV-Best1 GFP, which was capsid 7M8 (65).

### Injections.

Subretinal injections of approximately 0.3 μL AAV mixtures in PBS were made using pulled angled glass pipettes controlled by a Eppendorf Femtojet (Hamburg, Germany) as previously described (66). AAV8-hRedO-Nrf2, AAV8-CMV-Nrf2, and AAV8-hRedO-GFP-H2b was injected at 3 × 10^8^ genome copies (gc) per eye for each vector. AAV8-Best1-Nrf2 was tested with several different preparations, delivering from 2.10 × 10^8^ gc to 4.2 × 10^8^ gc per eye, and AAV27M8-Best1-GFP, which we used at 1 × 10^8^ gc per eye. We did not detect a difference in rescue between preparations or concentrations injected for AAV-Best1-Nrf2 for the ranges that we tested.

Our capsid and dosage choices were made based upon our experience with the toxicity of AAV preparations in the eye, which we previously reported (67). The RPE is sensitive to toxicity above a certain dose of AAVs, if those AAVs express in the RPE, with toxicity beginning at 5 × 10^8^ gc per eye. AAVs that use capsids that have a lower infectivity of the RPE, or AAVs that utilize photoreceptor-specific promoters (such as hRedO), cause significantly less toxicity to the RPE. We found, for instance, that at the highest doses tested (3 × 10^9^ gc/eye), photoreceptor-specific AAVs showed no toxicity to the RPE (67). For the majority of the present study, in the experimental eyes, we injected an AAV8 with a promoter active in the RPE (CMV or Best1) to drive expression of Nrf2, in combination with an AAV8 with a photoreceptor-specific promoter to express GFP in the photoreceptors for quantification of cones. The dose of AAV8 active in the RPE ranged from 2.10 × 10^8^ gc to 4.2 × 10^8^ gc per eye, below the threshold for toxicity (67). Most control eyes received a total dose of 3 × 10^8^ gc per eye of AAV8 hRedO-GFP-H2b. When AAV-Best1-Nrf2 and AAV-Best1-GFP were used in combination, we used a total dose of 3.1 × 10^8^ to 5.2 × 10^8^ per eye, with an AAV27M8 capsid for the AAV-Best1-GFP and an AAV8 capsid for the AAV-Best1-Nrf2. The AAV-27M8 capsid has a lower infectivity for the RPE and a lower toxicity to the RPE (16, 67).

### Histology.

For retinal and RPE whole mounts, enucleated eyes were processed as follows. The extraocular connective tissue and muscles were dissected from the eye, and then the RPE and retina were dissected apart for flat mounts. Tissue was fixed in 4% formaldehyde for 20 minutes at room temperature. For retinal sections, eyes were then fixed and cryoprotected in 5%, 15%, and 30% sucrose in PBS for a few hours and embedded in OCT on dry ice. Retinal sections or flat mounts were then blocked with 4% normal goat serum in PBS with 0.1% Triton X-100 for 1 hour at room temperature. Sections were incubated with primary antibodies in block solution at 4°C overnight, followed by secondary antibodies in PBS for 2 hours at room temperature. Flat mounts were incubated with primary antibodies for 2 days and with secondary antibodies for an additional 2 days. Primary antibodies included anti–cone arrestin (AB15282, MilliporeSigma), anti–glial fibrillary acidic protein (anti-GFAP; Z033401-2, DAKO), anti-GCLC (ab53179, Abcam), anti-MRP4 (ab15602, Abcam), and anti-TXNRD1 (ab16840, Abcam). Secondary antibodies include goat anti–rabbit Alexa Fluor 647 (A-21244, Thermo Fisher Scientific). During secondary incubation, tissue was incubated with Alexa Fluor 568 Phalloidin (A-12380, Thermo Fisher Scientific) to allow for imaging of the RPE cytoskeleton. Antibodies and phalloidin were diluted at 1:100.

*Imagingacquisition*. Tissue was coverslipped and imaged on a Nikon Ti inverted microscope with W1 Yokogawa spinning disk confocal using a 10× air, 20× air, or 40× oil objective. Preliminary image analysis was performed using Fiji (NIH) or Photoshop. A custom algorithm was created to detect GFP^+^ nuclei in retinal flat mounts to count cones. Briefly, this utilized Otsu’s method to binarize the nuclear cone signal, followed by a second pass to detect dimmer cones using a Laplacian of Gaussian method and, finally, fragmentation of merged objects into the number of cones based on an estimate of cone area (68). The algorithm was verified by hand-counting cones in multiple image slides. Cones were quantified across an entire retinal flat mount. Concentric circles were then drawn from the optic nerve head outwardly. The central one-third of the retina is most prone to degeneration during the time frame that we examined, whereas the peripheral cones often survive. Thus, we took the ratio of cones in the central one-third of the retina versus total number of cones in the flat mount to normalize for any variations in injection variability.

### Scanning EM.

The RPE and retina were quickly dissected apart from enucleated eyes and were fixed with 2.5% glutaraldehyde in 0.1M cacodylate buffer (pH 7.2), for 1–2 hours at room temperature. Samples were rinsed in 0.1M cacodylate buffer (pH 7.2) and then in distilled water, dehydrated in an ascending series of ethanol, and critical-point dried from liquid CO_2_ (Tousimis Autosamdri 815). Samples were then mounted on aluminum stubs with carbon conductive tabs and were sputter coated (Leica EM ACE600 sputter coater) with 5 nm platinum and observed in a field-emission scanning EM (Hitachi S-4700).

To quantify the relative surface areas of RPE cells with well-preserved apical microvilli, versus no apical processes, as well as areas of presumed RPE death, an ImageJ-based (NIH) automated method was used. Grayscale scanning EM photomicrographs were analyzed using 1000× magnification to minimize variability. Each 1000× scanning EM photomicrograph represented about 110 × 102 μm^2^ of surface area. In this study, we analyzed 13–33 scanning EM photomicrographs from each group, which included 14.5 × 10^4^ to 36.8 × 10^4^ μm^2^ of surface area at P30 and P45. Brightness and contrast were adjusted (Image → Adjust → Brightness/Contrast), and they were manually thresholded (Image → Adjust → Threshold) and converted to binary (Process → Binary) following the Histogram analysis (Analyze → Histogram). Black (level zero) pixels indicated areas with no apical processes, and white (level 255) pixels indicated microvilli. Areas of presumed RPE death were manually traced, and the number of pixels was calculated. The percentage of pixels that represented the relative surface areas of RPE cells with well-preserved microvillar membrane, RPE cells that exhibited no apical processes, and areas of RPE death were calculated as part of the total number of pixels.

*TransmissionEM*. Experimental mice were anesthetized with an i.p. injection of a ketamine-xylazine (100 mg/kg and 10 mg/kg, respectively) cocktail and were placed on a perfusion stage. Transcardial perfusion with 4% paraformaldehyde in HBSS was performed, and both eyes were enucleated. The extraocular connective tissue and muscles were dissected from the eyes, and the eyes were fixed with 2.5% glutaraldehyde in 0.1M cacodylate buffer (pH 7.2) for an additional 24 hours at 4°C. Following a triple rinse in cacodylate buffer, samples were postfixed with 1% osmium tetroxide/1.5% potassium ferrocyanide in 0.1M cacodylate buffer for 24 hours at room temperature in the dark. Samples were then washed 3 times in 0.1M cacodylate buffer (pH 7.2) and were then briefly washed in distilled water, dehydrated in an ascending series of ethanol concentrations, equilibrated in propylene oxide, and infiltrated and embedded in epoxy resin (Araldite 502/Embed-812 embedding media). Eyes were positioned in molds and polymerized in the oven at 60°C for 48 hours. The resin blocks were sectioned at 60–80 nm steps using a Reichert Ultracut S ultramicrotome. The sections were mounted on copper Formvar/Carbon-coated grids and viewed with a JEOL 1200EX microscope operating at 80 kV. Images were captured with an Advanced Microscopy Techniques camera system at 3488 × 2580 pixel resolution.

### Optomotor.

Visual acuity was determined using an OptoMotry System (CerebralMechanics Inc.) as previously described (69). All tests were performed between 9 a.m. and 2 p.m. by an experienced technician blinded to the treatment groups. Mice were placed on the platform and allowed to habituate to the chamber for 5 minutes. They were then assessed for reflexive head-tracking movements in concert with the rate of a virtual sine wave grating on the surrounding monitors, and the visual acuity threshold was determined at the highest spatial frequency (cycle/degree) when the animal stopped tracking. Testing was done with a grating of a 12°/second drifting speed and 100% contrast. The right and left eyes were tested independently, as they responded separately to counterclockwise and clockwise grating rotations, respectively (70). A staircase procedure was used, in which the observer tested low to high visual acuity. Each animal was tested for 5–15 minutes per session. Testing took place between P35 and P60.

*Transcriptomeprofiling*. The transcriptome profiling experiments were designed to maximize the use of paired comparisons between eyes of the same mouse, to decrease variation seen in degeneration or genetic background. Thus, each mouse had one eye injected with AAV-Best1-Nrf2 and AAV-Best1-GFP, and the contralateral eye injected with AAV-Best1-GFP only. Immediately after sacrifice, the eye was enucleated and the level of GFP infection was examined to verify completeness of infection. The retina was then removed, and the eyecup was quickly cleaned of connective tissue and placed in RNA Protect (Qiagen). The supernatant was then collected and purified with a RNAeasy micro column (Qiagen), adapting the protocol introduced by Xin-Zhao Wang et al. (71). Purified RPE RNA was then amplified with a Ultra Low v4 kit (Clontech, Takara Bio), and libraries were made for Illumina sequencing. A total of 6 paired replicates were used for the P20 *rd1* comparison, 4 paired replicates were used for the P40 *rd1* comparison, and 5 paired replicates were used for the P40 CD1 comparison. All samples were processed using an RNA sequencing pipeline implemented in the bcbio-nextgen project (https://bcbio-nextgen.readthedocs.org/en/latest/). Raw reads were examined for quality issues using FastQC (http://www.bioinformatics.babraham.ac.uk/projects/fastqc/) to ensure library generation and sequencing were suitable for further analysis.

Raw reads were aligned to UCSC build mm10 of the mouse genome augmented with transcript information from Ensembl release 91 using STAR (72). Alignments were checked for evenness of coverage, rRNA content, genomic context of alignments (for example, alignments in known transcripts and introns), complexity, and other quality checks using a combination of FastQC, Qualimap, MultiQC (73), and custom tools (74). Counts of reads aligned to known genes were generated by featureCounts (75). In parallel, transcripts per million (TPM) measurements per isoform were generated by quasialignment using Salmon (76). Differential expression at the gene level was performed with DESeq2 using the counts per gene estimated from the Salmon quasialignments brought into DESeq2 by tximport (77, 78). Lists of differentially expressed genes were examined for gene ontology (GO) and KEGG term enrichment with clusterProfiler (79). In addition, a cut-off–free gene set enrichment analysis (GSEA) was performed using clusterProfiler and weighted fold change calculations from DESeq2. UpSet plots of shared differentially expressed genes, and GO terms between analyses were generated using the UpSetR R package (80). The raw results have been deposited to the GEO repository (GEO161106; https://www.ncbi.nlm.nih.gov/geo/query/acc.cgi?acc=GSE161106).

### Statistics.

Statistical analyses of cone ratios were conducted using 2-tailed paired *t* test. A *P* value of less than 0.05 was considered statistically significant. Statistical analysis of optomotor visual acuity were conducted by 2-tailed paired *t* test. A *P* value of less than 0.05 was considered statistically significant. Statistical analysis of RPE surface area with microvilli coverage were performed by 1-way ANOVA. A *P* value of less than 0.05 was considered statistically significant. Detailed statistical analyses for transcriptome analyses are specifically described in the methods for transcriptome profiling.

*Studyapproval*. All experimental procedures were approved by the IACUC at Harvard University and adhered to the ARVO Statement for the Use of Animals in Ophthalmic and Vision Research.

## Author contributions

DMW and CLC designed the studies. DMW, XJ, MVI, M. Chung, SKW, YX, HX, SRZ, and PR conducted experiments and acquired data. DMW, XJ, MVI, M. Chung, MP, EW, WX, and CLC analyzed data. DMW, MVI, M. Cicconet, and CLC provided reagents. DMW, MVI, MP, WX, and CLC wrote the manuscript.

## Supplementary Material

Supplemental data

## Version 1 01/25/2021

Electronic publication
