# Supplementary material for: Nrf2 overexpression rescues the RPE in mouse models of retinitis pigmentosa
Source: JCI Insight. 2021 Jan 25;6(2):e145029. doi: 10.1172/jci.insight.145029 (PMC7934854; doi:10.1172/jci.insight.145029)
Supplement: Supplemental data [file jciinsight-6-145029-s248.pdf]

Supplemental Table 1

Top 30 GO Biological Processes

Wild type P40

	ID	Description	Gene Ratio	BgRatio	Pvalue	Padjust
1	GO:0002443	leukocyte mediated immunity	79/1648	354/20604	3.05E-17	1.72E-13
2	GO:0006749	glutathione metabolic process	26/1648	49/20604	2.38E-16	6.73E-13
3	GO:0006909	phagocytosis	48/1648	175/20604	1.48E-14	2.78E-11
4	GO:0002250	adaptive immune response	81/1648	412/20604	2.45E-14	3.46E-11
5	GO:0002460	adaptive immune response based on somatic recombination of immune receptors built from immunoglobulin superfamily domains	62/1648	289/20604	5.26E-13	5.33E-10
6	GO:0002274	myeloid leukocyte activation	48/1648	192/20604	6.57E-13	5.33E-10
7	GO:0051186	cofactor metabolic process	85/1648	470/20604	7.25E-13	5.33E-10
8	GO:0048002	antigen processing and presentation of peptide antigen	26/1648	64/20604	7.54E-13	5.33E-10
9	GO:0002449	lymphocyte mediated immunity	59/1648	271/20604	9.96E-13	6.25E-10
10	GO:0002694	regulation of leukocyte activation	87/1648	496/20604	2.21E-12	1.25E-09
11	GO:0002703	regulation of leukocyte mediated immunity	53/1648	234/20604	2.81E-12	1.45E-09
12	GO:0002699	positive regulation of immune effector process	53/1648	243/20604	1.32E-11	6.20E-09
13	GO:0002697	regulation of immune effector process	72/1648	390/20604	1.57E-11	6.83E-09
14	GO:0019882	antigen processing and presentation	31/1648	100/20604	2.15E-11	8.66E-09
15	GO:0050867	positive regulation of cell activation	62/1648	318/20604	3.95E-11	1.49E-08
16	GO:0006575	cellular modified amino acid metabolic process	40/1648	160/20604	5.41E-11	1.91E-08
17	GO:0050900	leukocyte migration	62/1648	325/20604	1.01E-10	3.37E-08
18	GO:0032103	positive regulation of response to external stimulus	57/1648	287/20604	1.14E-10	3.59E-08
19	GO:0016053	organic acid biosynthetic process	63/1648	339/20604	2.21E-10	6.58E-08
20	GO:0002696	positive regulation of leukocyte activation	58/1648	304/20604	4.04E-10	1.14E-07
21	GO:0046394	carboxylic acid biosynthetic process	62/1648	338/20604	5.30E-10	1.43E-07
22	GO:0006790	sulfur compound metabolic process	52/1648	260/20604	5.74E-10	1.47E-07
23	GO:0072593	reactive oxygen species metabolic process	54/1648	276/20604	6.25E-10	1.53E-07
24	GO:0099024	plasma membrane invagination	21/1648	56/20604	7.11E-10	1.67E-07
25	GO:0030595	leukocyte chemotaxis	45/1648	210/20604	8.32E-10	1.88E-07
26	GO:0060326	cell chemotaxis	56/1648	295/20604	9.81E-10	2.13E-07
27	GO:0010324	membrane invagination	22/1648	63/20604	1.37E-09	2.84E-07
28	GO:0002705	positive regulation of leukocyte mediated immunity	38/1648	163/20604	1.41E-09	2.84E-07
29	GO:0002819	regulation of adaptive immune response	42/1648	193/20604	1.85E-09	3.60E-07
30	GO:0002683	negative regulation of immune system process	74/1648	453/20604	2.68E-09	5.05E-07

rd1 P20

	ID	Description	Gene Ratio	BgRatio	Pvalue	Padjust
1	GO:0050953	sensory perception of light stimulus	53/2024	144/19631	2.02E-17	7.48E-14
2	GO:0007601	visual perception	52/2024	140/19631	2.56E-17	7.48E-14
3	GO:0006749	glutathione metabolic process	23/2024	49/19631	7.29E-11	1.42E-07
4	GO:0060041	retina development in camera-type eye	43/2024	146/19631	1.09E-10	1.59E-07
5	GO:0001505	regulation of neurotransmitter levels	78/2024	367/19631	3.72E-10	4.23E-07
6	GO:0051186	cofactor metabolic process	93/2024	470/19631	4.35E-10	4.23E-07
7	GO:0009636	response to toxic substance	68/2024	318/19631	3.80E-09	2.81E-06
8	GO:0009583	detection of light stimulus	21/2024	49/19631	3.85E-09	2.81E-06
9	GO:0043523	regulation of neuron apoptotic process	57/2024	254/19631	1.13E-08	6.71E-06
10	GO:0051402	neuron apoptotic process	61/2024	280/19631	1.15E-08	6.71E-06
11	GO:0007602	phototransduction	15/2024	28/19631	1.53E-08	8.10E-06
12	GO:0042461	photoreceptor cell development	21/2024	53/19631	2.09E-08	1.01E-05
13	GO:0050806	positive regulation of synaptic transmission	46/2024	193/19631	4.27E-08	1.91E-05
14	GO:0051188	cofactor biosynthetic process	51/2024	225/19631	4.67E-08	1.95E-05
15	GO:1990748	cellular detoxification	14/2024	28/19631	1.45E-07	5.65E-05
16	GO:0048167	regulation of synaptic plasticity	46/2024	204/19631	2.46E-07	8.97E-05
17	GO:0050804	modulation of chemical synaptic transmission	88/2024	499/19631	3.31E-07	0.00011376
18	GO:0099177	regulation of trans-synaptic signaling	88/2024	500/19631	3.62E-07	0.00011749
19	GO:1901214	regulation of neuron death	67/2024	351/19631	4.75E-07	0.00014588
20	GO:0098754	detoxification	16/2024	39/19631	5.72E-07	0.00016702
21	GO:0042391	regulation of membrane potential	77/2024	427/19631	7.33E-07	0.00020376
22	GO:0005996	monosaccharide metabolic process	51/2024	247/19631	1.00E-06	0.00025824
23	GO:0042462	eye photoreceptor cell development	15/2024	36/19631	1.02E-06	0.00025824
24	GO:0010035	response to inorganic substance	77/2024	431/19631	1.07E-06	0.00025915
25	GO:0070997	neuron death	71/2024	389/19631	1.25E-06	0.00027743
26	GO:0072593	reactive oxygen species metabolic process	55/2024	276/19631	1.25E-06	0.00027743
27	GO:0006836	neurotransmitter transport	58/2024	297/19631	1.28E-06	0.00027743
28	GO:0010038	response to metal ion	53/2024	263/19631	1.36E-06	0.00028256
29	GO:0046530	photoreceptor cell differentiation	22/2024	72/19631	1.90E-06	0.00038326
30	GO:0009584	detection of visible light	13/2024	30/19631	3.17E-06	0.00061582

rd1 P40

	ID	Description	Gene Ratio	BgRatio	Pvalue	Padjust
1	GO:0050953	sensory perception of light stimulus	48/1315	144/20604	2.19E-22	1.14E-18
2	GO:0007601	visual perception	47/1315	140/20604	4.27E-22	1.14E-18
3	GO:0006749	glutathione metabolic process	21/1315	49/20604	4.78E-13	6.40E-10
4	GO:0009583	detection of light stimulus	21/1315	49/20604	4.78E-13	6.40E-10
5	GO:0007602	phototransduction	14/1315	28/20604	2.99E-10	3.21E-07
6	GO:0006575	cellular modified amino acid metabolic process	33/1315	160/20604	1.65E-09	1.48E-06
7	GO:0009582	detection of abiotic stimulus	28/1315	122/20604	2.28E-09	1.74E-06
8	GO:0009581	detection of external stimulus	28/1315	123/20604	2.77E-09	1.86E-06
9	GO:0046942	carboxylic acid transport	47/1315	294/20604	5.17E-09	3.08E-06
10	GO:0015849	organic acid transport	47/1315	295/20604	5.78E-09	3.10E-06
11	GO:0009584	detection of visible light	13/1315	30/20604	1.18E-08	5.76E-06
12	GO:0009636	response to toxic substance	50/1315	334/20604	1.58E-08	7.05E-06
13	GO:0006790	sulfur compound metabolic process	42/1315	260/20604	2.47E-08	1.02E-05
14	GO:0051186	cofactor metabolic process	62/1315	470/20604	4.26E-08	1.63E-05
15	GO:0043523	regulation of neuron apoptotic process	40/1315	255/20604	1.20E-07	4.21E-05
16	GO:0015711	organic anion transport	54/1315	398/20604	1.26E-07	4.21E-05
17	GO:0051187	cofactor catabolic process	16/1315	55/20604	1.92E-07	6.05E-05
18	GO:0051402	neuron apoptotic process	42/1315	282/20604	2.53E-07	7.54E-05
19	GO:0034764	positive regulation of transmembrane transport	35/1315	217/20604	3.67E-07	0.00010075
20	GO:0072593	reactive oxygen species metabolic process	41/1315	276/20604	3.76E-07	0.00010075
21	GO:1901214	regulation of neuron death	48/1315	352/20604	5.37E-07	0.00013712
22	GO:0050890	cognition	44/1315	311/20604	5.73E-07	0.00013947
23	GO:0032147	activation of protein kinase activity	37/1315	242/20604	6.73E-07	0.00015676
24	GO:0042461	photoreceptor cell development	16/1315	60/20604	7.12E-07	0.00015899
25	GO:0006911	phagocytosis	14/1315	47/20604	8.08E-07	0.00016995
26	GO:0048871	multicellular organismal homeostasis	58/1315	466/20604	8.25E-07	0.00016995
27	GO:0034765	regulation of ion transmembrane transport	56/1315	453/20604	1.58E-06	0.00030619
28	GO:0006979	response to oxidative stress	51/1315	398/20604	1.60E-06	0.00030619
29	GO:0070997	neuron death	50/1315	391/20604	2.14E-06	0.00039613
30	GO:0009416	response to light stimulus	40/1315	285/20604	2.26E-06	0.00040358

Untreated

	ID	Description	Gene Ratio	BgRatio	Pvalue	Padjust
1	GO:0033108	mitochondrial respiratory chain complex assembly	45/3196	74/19631	5.64E-18	3.37E-14
2	GO:0022613	ribonucleoprotein complex biogenesis	129/3196	402/19631	2.07E-15	6.19E-12
3	GO:0010257	NADH dehydrogenase complex assembly	29/3196	43/19631	9.01E-14	1.35E-10
4	GO:0032981	mitochondrial respiratory chain complex I assembly	29/3196	43/19631	9.01E-14	1.35E-10
5	GO:0007005	mitochondrion organization	132/3196	443/19631	5.13E-13	6.13E-10
6	GO:0042254	ribosome biogenesis	88/3196	260/19631	2.34E-12	2.33E-09
7	GO:0016072	rRNA metabolic process	75/3196	209/19631	3.85E-12	3.28E-09
8	GO:0006364	rRNA processing	67/3196	179/19631	5.99E-12	4.48E-09
9	GO:0034470	ncRNA processing	102/3196	327/19631	1.21E-11	8.03E-09
10	GO:0022900	electron transport chain	38/3196	86/19631	9.59E-10	5.73E-07
11	GO:0022904	respiratory electron transport chain	36/3196	80/19631	1.43E-09	7.75E-07
12	GO:0006119	oxidative phosphorylation	39/3196	91/19631	1.72E-09	8.07E-07
13	GO:0034660	ncRNA metabolic process	121/3196	442/19631	1.76E-09	8.07E-07
14	GO:0008380	RNA splicing	101/3196	354/19631	3.72E-09	1.59E-06
15	GO:0042775	mitochondrial ATP synthesis coupled electron transport	28/3196	57/19631	8.52E-09	3.39E-06
16	GO:0042773	ATP synthesis coupled electron transport	29/3196	61/19631	1.20E-08	4.47E-06
17	GO:0022618	ribonucleoprotein complex assembly	67/3196	211/19631	1.78E-08	6.26E-06
18	GO:0071826	ribonucleoprotein complex subunit organization	69/3196	224/19631	4.27E-08	1.42E-05
19	GO:0045333	cellular respiration	53/3196	157/19631	5.71E-08	1.80E-05
20	GO:0032543	mitochondrial translation	31/3196	72/19631	6.88E-08	2.06E-05
21	GO:0006397	mRNA processing	112/3196	432/19631	1.64E-07	4.68E-05
22	GO:0009141	nucleoside triphosphate metabolic process	76/3196	272/19631	7.54E-07	0.00020482
23	GO:0009205	purine ribonucleoside triphosphate metabolic process	69/3196	248/19631	2.81E-06	0.00073062
24	GO:0140053	mitochondrial gene expression	34/3196	96/19631	3.91E-06	0.00097314
25	GO:0015980	energy derivation by oxidation of organic compounds	68/3196	246/19631	4.22E-06	0.00100958
26	GO:0009144	purine nucleoside triphosphate metabolic process	70/3196	256/19631	4.65E-06	0.00106944
27	GO:0009199	ribonucleoside triphosphate metabolic process	69/3196	252/19631	5.15E-06	0.00114065
28	GO:0000375	RNA splicing	66/3196	239/19631	6.01E-06	0.00119821
29	GO:0000377	RNA splicing	66/3196	239/19631	6.01E-06	0.00119821
30	GO:0000398	mRNA splicing	66/3196	239/19631	6.01E-06	0.00119821

Supplemental Figure 1. Plot showing the number of cone photoreceptors counted in the central retina of P40-P60 mice, where the majority of degeneration takes place, as a ratio of total number of cone photoreceptors in a retina (to normalize for any variations in injection and infection efficiency across different retinas). Data shown as mean ± s.d. (*n* = 9 mice).

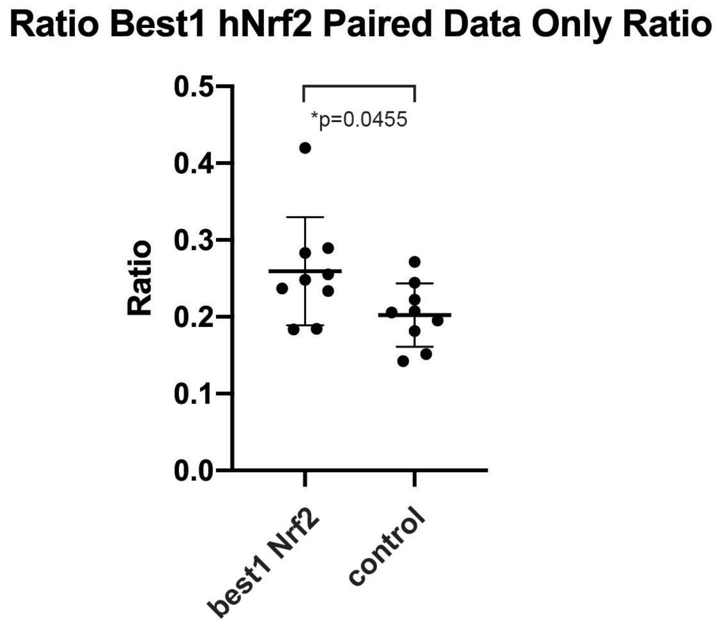

Supplemental Figure 2. KEGG pathways for glutathione metabolism for each comparison. Enzymes in red have increased expression levels whereas enzymes in green have decreased expression level. For Nrf2 treated tissue across all three comparisons (P20 *rd1* Nrf2 treated vs untreated, P40 *rd1* Nrf2 treated vs untreated, wild type Nrf2 treated vs untreated) there was upregulation of several enzymes in the glutathione pathway (in red). These enzymes were downregulated when comparing P40 to P20 untreated *rd1* (green).

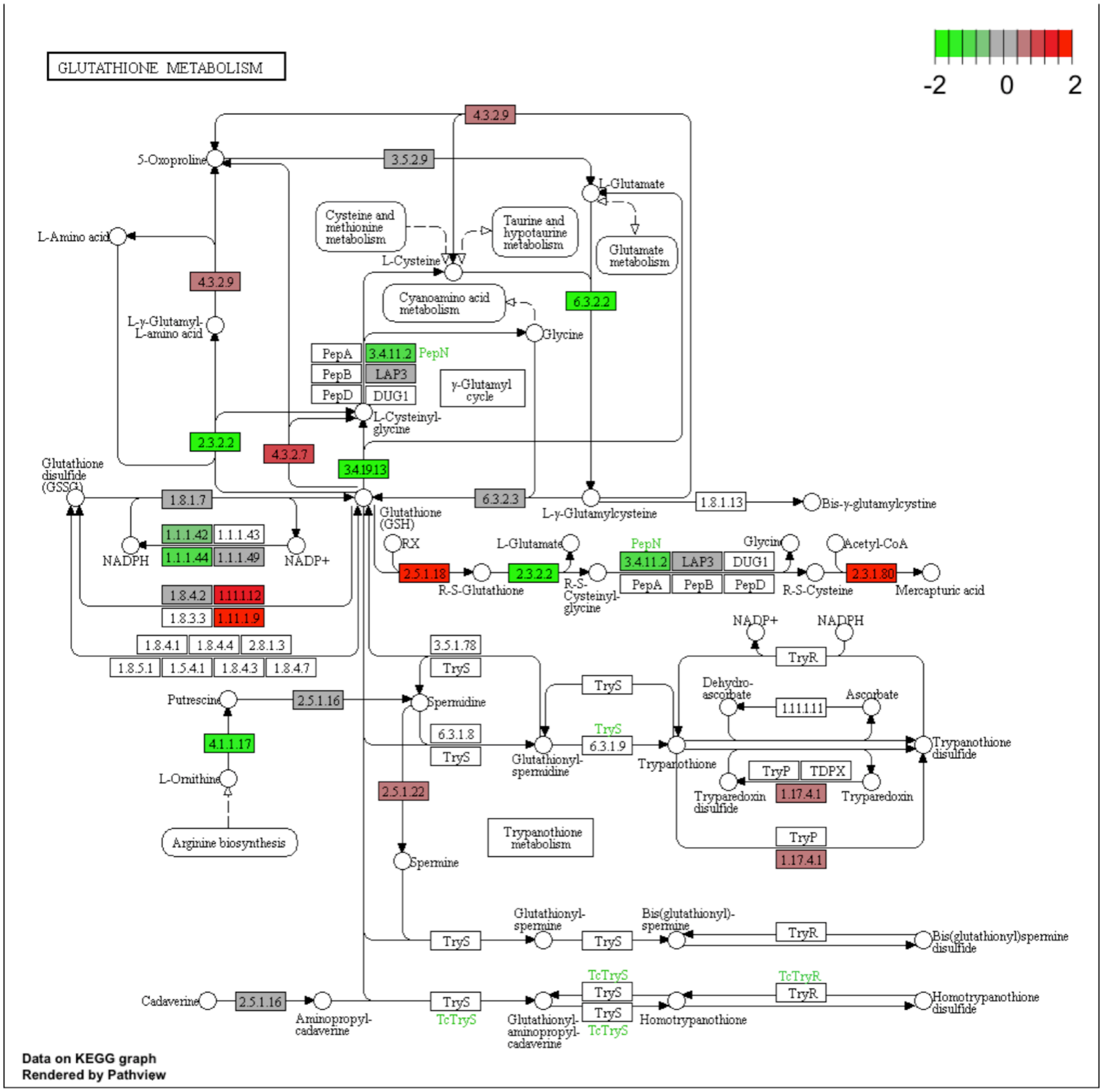

Untreated *rd1* p40 vs *rd1* p20

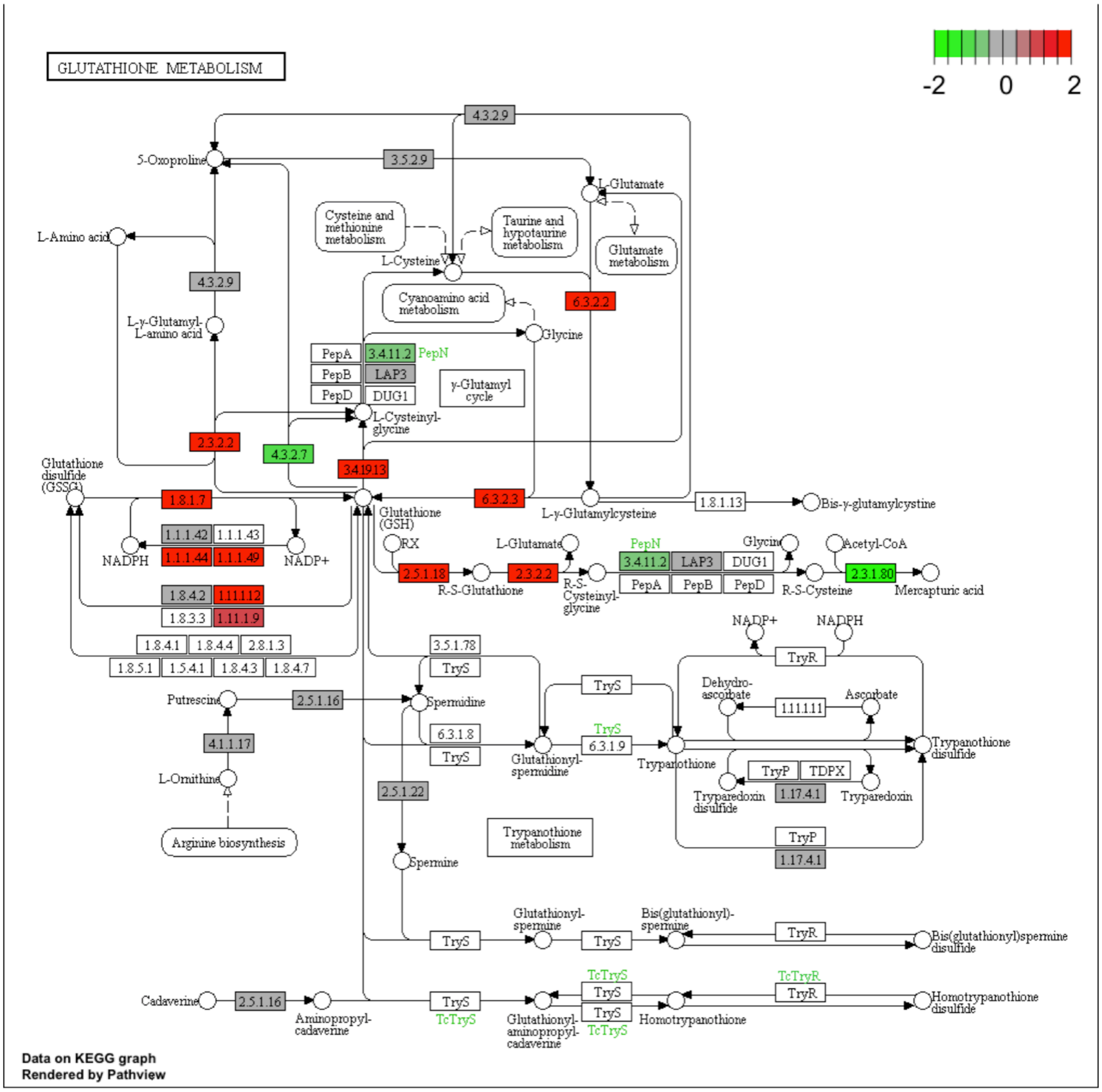

P20 Nrf2 in *rd1* vs untreated *rd1*

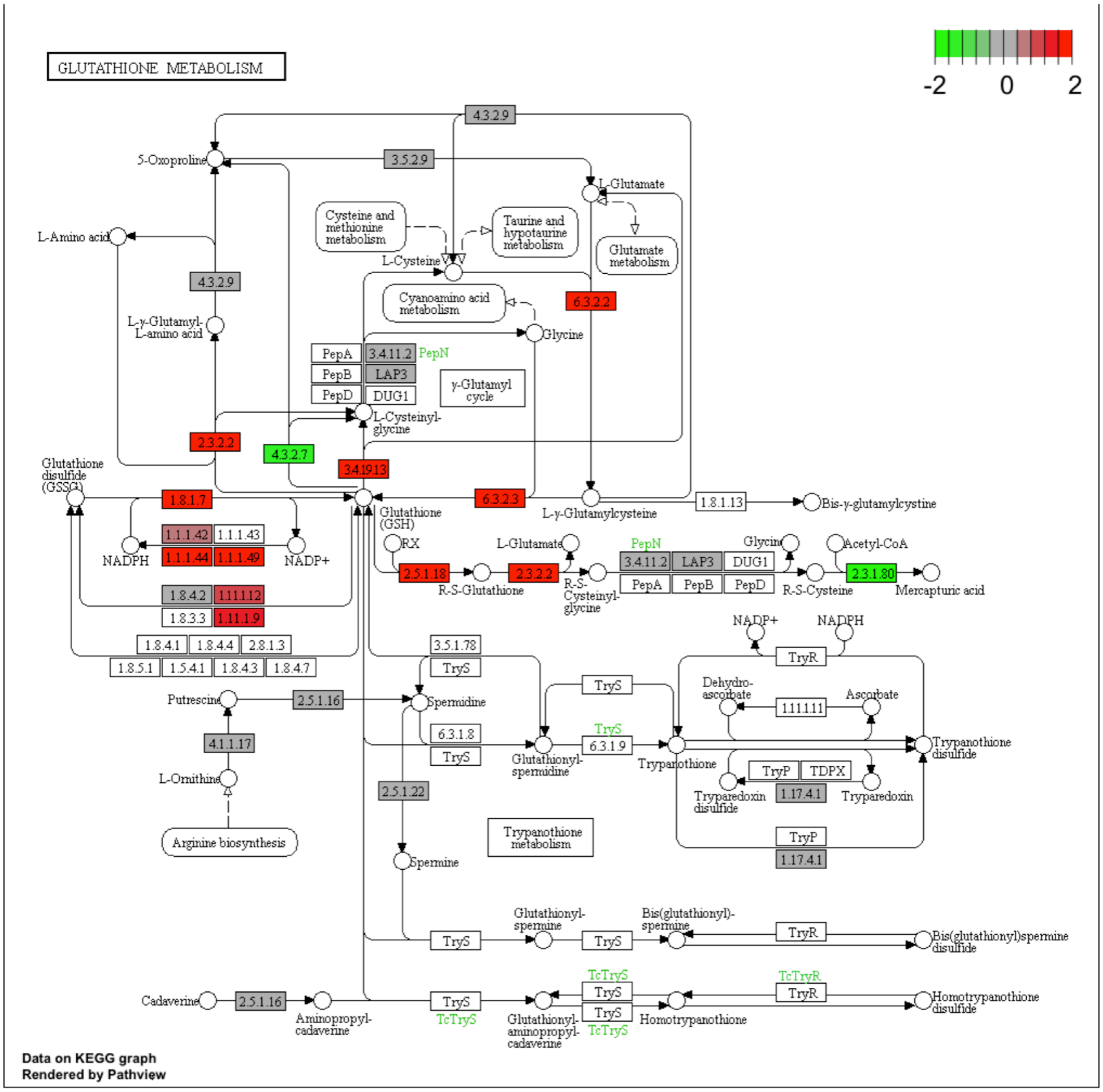

P40 Nrf2 in *rd1* vs untreated *rd1*

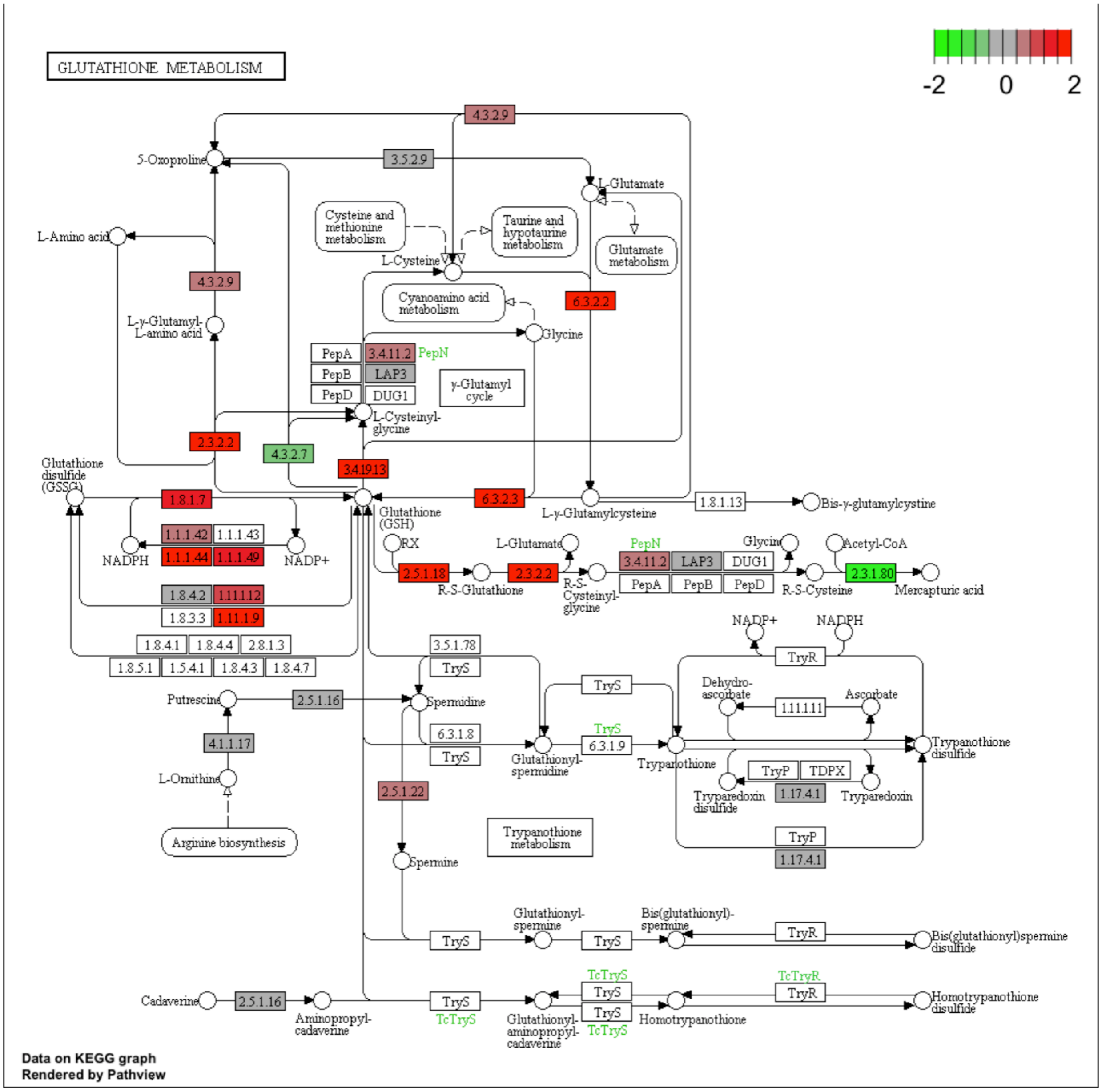

P40 wild type
